# High flux novel polymeric membrane for renal applications

**DOI:** 10.1038/s41598-023-37765-y

**Published:** 2023-07-20

**Authors:** Christa N. Hestekin, Efecan Pakkaner, Jamie A. Hestekin, Leticia Santos De Souza, Partha Pratim Chowdhury, Juliana Louzada Marçal, John Moore, Sarah A. Hesse, Christopher J. Takacs, Christopher J. Tassone, Soma Shekar Dachavaram, Peter A. Crooks, Kate Williams, Ira Kurtz

**Affiliations:** 1 Ralph E. Martin Department of Chemical Engineering, University of Arkansas, 3202 Bell Engineering Center, Fayetteville, AR 72701 USA; 2 Stanford Synchrotron Radiation Lightsource, SLAC National Accelerator Laboratory, Menlo Park, CA 94025 USA; 3 Department of Pharmaceutical Sciences, College of Pharmacy, University of Arkansas for Medical Sciences, Little Rock, AR 72205 USA; 4 St. Francis Animal Hospital, 121 Virginia Street, Springdale, AR 72764 USA; 5 Division of Nephrology, Department of Medicine, David Geffen School of Medicine, University of California, Los Angeles, CA 90095 USA; 6 Brain Research Institute, University of California, Los Angeles, CA 90095 USA

**Keywords:** Nephrology, Biomedical engineering

## Abstract

Biocompatibility and the ability to mediate the appropriate flux of ions, urea, and uremic toxins between blood and dialysate components are key parameters for membranes used in dialysis. Oxone-mediated TEMPO-oxidized cellulose nanomaterials have been demonstrated to be excellent additives in the production and tunability of ultrafiltration and dialysis membranes. In the present study, nanocellulose ionic liquid membranes (NC-ILMs) were tested in vitro and ex vivo. An increase in flux of up to two orders of magnitude was observed with increased rejection (about 99.6%) of key proteins compared to that of polysulfone (PSf) and other commercial membranes. NC-ILMs have a sharper molecular weight cut-off than other phase inversion polymeric membranes, allowing for high throughput of urea and a uremic toxin surrogate and limited passage of proteins in dialysis applications. Superior anti-fouling properties were also observed for the NC-ILMs, including a > 5-h operation time with no systemic anticoagulation in blood samples. Finally, NC-ILMs were found to be biocompatible in rat ultrafiltration and dialysis experiments, indicating their potential clinical utility in dialysis and other blood filtration applications. These superior properties may allow for a new class of membranes for use in a wide variety of industrial applications, including the treatment of patients suffering from renal disease.

## Introduction

Hemodialysis (HD) plays an important role in providing life support for patients with end-stage renal disease (ESRD). According to the United States Renal Data System 2020 Annual Data Report, in the U.S. alone, there are approximately 600,000 patients undergoing long-term treatment on hemodialysis (https://www.niddk.nih.gov/health-information/health-statistics/). In addition, patients are temporarily treated with HD when they have acute kidney injury (AKI). In HD, blood and an electrolyte solution (dialysate) are brought into contact in a dual-chambered filter called a dialyzer. A semi-permeable membrane within the dialyzer allows waste products in the blood to diffuse into the dialysate, leaving behind cells and larger proteins in the blood. Various polymeric materials have been used as dialysis membranes, including cellulose acetate (CA)^1^, polyacrylonitrile (PAN)^2^, polysulfone (PSf)^3^ and others, which vary in their flux properties. A membrane is considered acceptable for dialysis applications based on its biocompatibility and ability to mediate the flow of ions, urea, and uremic toxins between the patient’s bloodstream and the dialysate solution within the timeframe of a typical treatment session.

In a conventional dialyzer, two different modes of operation can be utilized. In one mode, the membrane acts as an ultrafiltration system, in that a hydrostatic pressure gradient is applied. In this mode of operation, various amounts of water can be removed depending on the patient’s volume status. In the other mode of operation is a diffusion-driven process where, in the absence of a pressure graduate between the two streams, a chemical potential gradient (µ) induces the permeation of a solute through the membrane. Various ions, urea and uremic toxins permeate in this manner.

In addition to dialysis, ultrafiltration membranes have had a wide range of successful applications which include drinking water treatment^4^, endotoxin and pyrogenic removal^5^, and separation of micropollutants^6^. However, in all applications, fouling of membranes remains a challenge that adversely impacts the membrane performance^4^. Blood components create a particularly significant challenge during dialysis, where proteins, clotting factors and cells can significantly affect the ultrafiltration properties^7,8^. To boost the anti-fouling properties of a typical ultrafiltration membrane, modifications to surface properties (e.g., hydrophilicity^9,10^, negative surface charge density^9,10^ and coupling with anticoagulants) have been attempted^11^. Furthermore, a variety of modified surface coatings, such as poly-dopamine^12^, heparin^7^, chitosan^13^, poly l-lysine^14^ and mucin^15^, have been utilized. Although these have been employed with some success, a long-term remedy to sufficiently increase the biocompatibility of membranes with blood has not yet been achieved. Even a membrane with high flux and low fouling may not be a good candidate for dialysis. An additional important parameter for assessing dialysis membranes is complement complex activation, which can result in clotting^16–18^, swelling^19–21^, and other detrimental effects, serving as an indicator of the body’s immune response to a foreign substance. It has been found that certain membrane materials, such as polyacrylonitrile, cause an immune response, while others (including cellulose triacetate and PSf) do not^22^.

Cellulose derivatives have been utilized in dialysis membranes^23,24^. However, cellulose in its pure form is difficult to process because of its low solubility in water and many organic solvents. TEMPO oxidized cellulose contains carboxylate groups which make the material more hydrophilic, thereby improving solubility. The TEMPO oxidation process has been explored previously^25–28^. Although TEMPO oxidized cellulose produces materials that are present in crystalline nanofibrils that form larger fibrous structures in the native form, modifying these biopolymers via dissolution and precipitation is possible^29^. TEMPO oxidized cellulose has also been used to modify ultrafiltration membranes^28,30^.

To addresses the challenge of dissolving cellulose, ionic liquids have been explored as alternative solvents.^31,32^ In this study, novel nanocrystalline ionic liquid membranes (NC-ILMs) were developed using phase inversion of TEMPO oxidized cellulose dissolved in ionic liquids with water as an anti-solvent. During this process, carboxylate groups were introduced on the cellulose surface by the Oxone-mediated surface oxidation through controlled reaction conditions replacing the hydroxyl groups. The ionic interactions of the carboxylate groups with the ionic liquid are hypothesized to play a critical role in the phase inversion process and in turn the membrane structure and properties. To our knowledge, this is the first time this technique has been used to successfully develop membranes with superior ultrafiltration and dialysis fluxes. Furthermore, these membranes show unique structural properties as demonstrated by scanning electron microscopy (SEM) and wide-angle X-ray scattering (WAXS). This paper outlines the uniqueness and superior functioning capabilities of these novel membranes.

## Results

**Figure 1:**
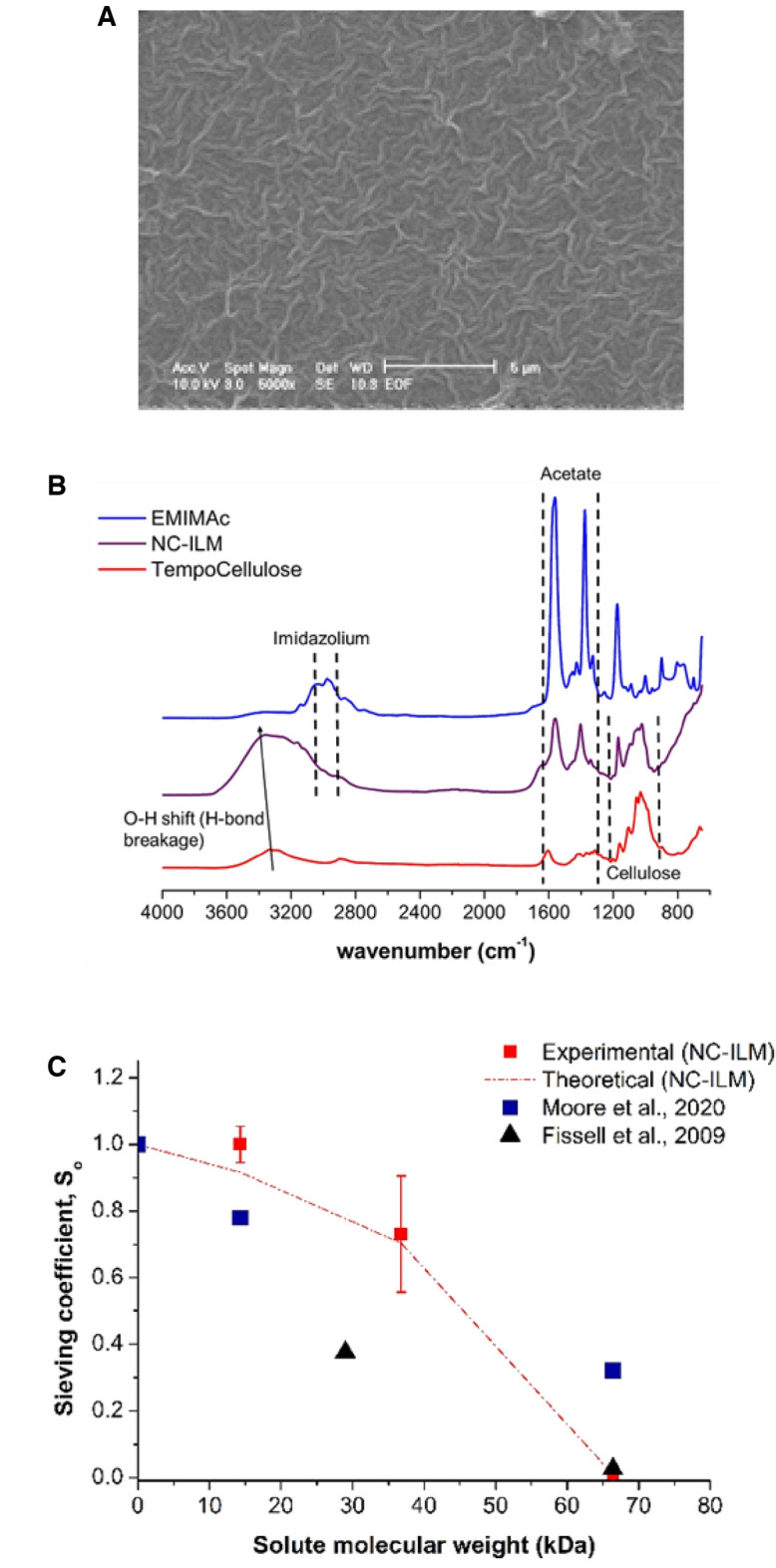
General characterization of NC-ILM. (**A**) SEM imaging of final NC-ILM membrane (scale: 5 µm). (**B**) FT-IR spectra of TEMPO Oxidized cellulose, 1-ethyl-3-methylimidazolium acetate (EMIMAc) ionic liquid and final NC-ILM product directly after casting. (**C**) Sieving coefficients plotted for experimental (markers) and theoretical (line) for the NC-ILMs for varying molecular weight proteins obtained during in vitro experiments.

The NC-ILMs were made via phase inversion through the process shown in Fig. S-1. While a typical polymeric phase inversion process produces a continuous phase membrane, as shown in Fig. 1A, this material appears to have interconnected fibers like what has previously been observed in electrospun membranes^33^. However, unlike a typical electrospun membrane, this new membrane appears to have an ordered arrangement. This new membrane was analyzed using WAXS before and after the phase inversion process, shown in Figs. S-2 and S-3, respectively. The NC-ILM WAXS results demonstrate that cellulose is well dissolved by the ionic liquid (IL) and the phase inverted membranes do not contain conventional crystalline cellulose.

X-ray imaging was performed on the NC-ILM and compared to PSf membranes produced by the same non-solvent induced phase inversion process. As shown in Figs. S-4 and S-5, the X-ray micrographs of the PSf phase inverted material show fine features of small pores visible near the top surface (right side of the image) of the membrane and larger, coarser features evolving towards the bottom (left side of the image) of the membrane. This structure is absent in images of the NC-ILM in normal incidence as shown in Fig. S-6. The membrane edge (bottom of the image) shows no obvious large-scale structure that is associated with typical phase inverted membranes. Qualitatively, the NC-ILM is less rigid with a consistency that is reminiscent of a gel. With the exception of a few small cracks, there is no measurable internal structure of the NC-ILM from imaging, suggesting that the internal structure has very little electron density fluctuations on a scale of > 3 microns. These observations are consistent with the optical clarity of the NC-ILM compared to the white appearance of the PSf membrane due to the strong visible light scattering.

Using the Guerout–Elford–Ferry method^34^ (equations in Supplemental Section S-1) the average pore size is 12.5 ± 1.5 nm which is larger than dialysis membranes reported in literature (comparison between cellulosic and silicon slit membranes is shown in Table S-1). Previous studies have reported active layer thicknesses of phase inversion membranes that were very constant and ranged from 0.027 to 17.5 µm with PSf, depending on the casting conditions^35^. The NC-ILM has an active layer (estimated from Figs. S-7 and S-8) of 2.39 and 1.1 µm as measured by Image J analysis. As shown in Fig. 1B, acetate groups are present in the final membrane structure immediately after casting, even after rinsing. However, as Fig. S-9 shows, acetate is removed after a methanol soak and no changes in chemistry were observed over the following 30 days.

**Figure 2:**
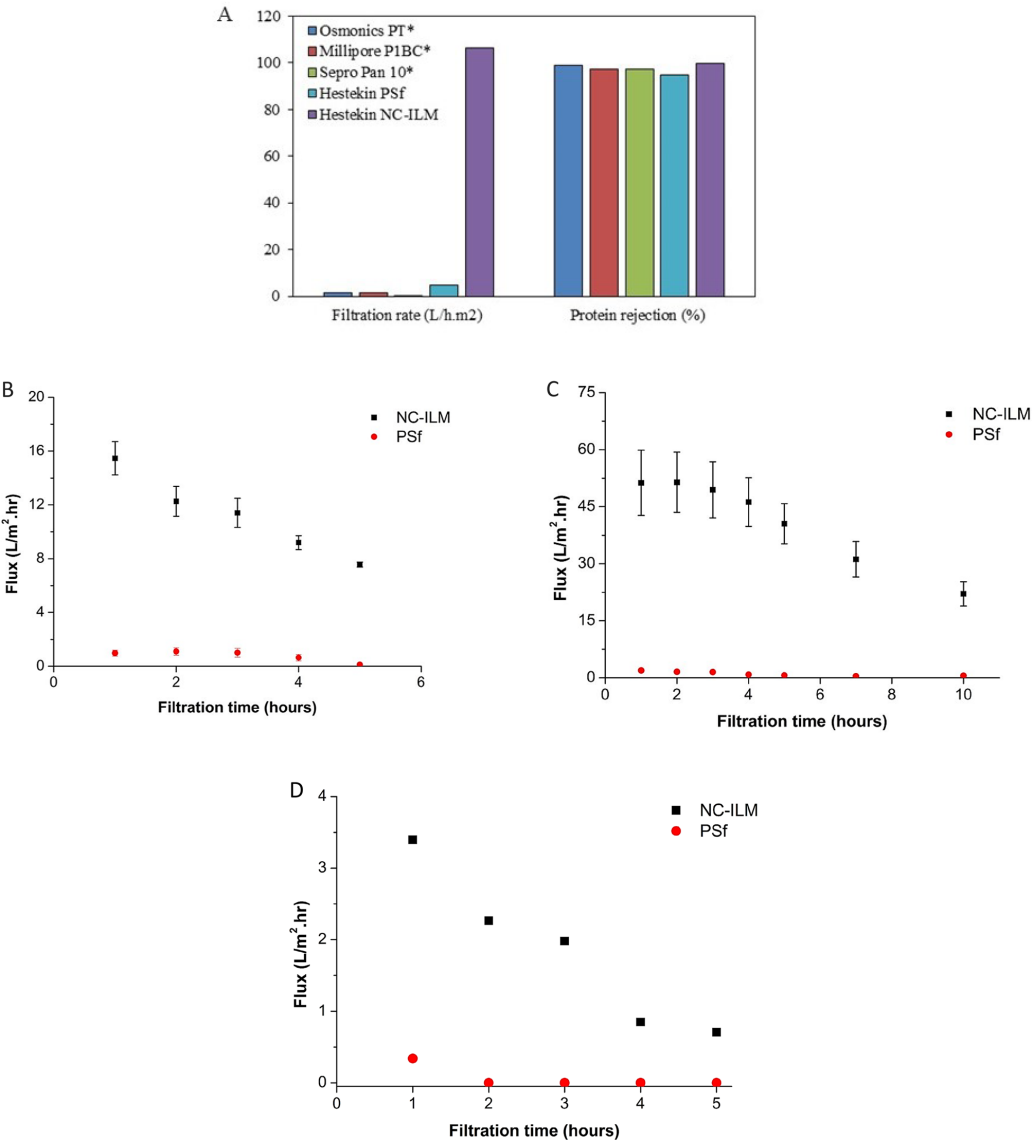
Comparison between different membrane performances. (**A**) BSA rejection abilities between commercial membranes and lab made membranes (P = 40 psig). (**B**) Change in the average permeate flux of heparinized whole porcine blood through NC-ILM and PSf membranes (n = 3). (**C**) Change in the average permeate flux of citrated whole porcine blood through NC-ILM and PSf membranes (n = 3). (**D**) Change in permeate flux of non-anticoagulated whole porcine blood through NC-ILM and PSf (n = 1).

The sieving coefficient of this membrane is plotted versus solute molecular weight and compared to previously-characterized porous^28^ and slit membranes^28,36,37^ in Fig. 1C. The NC-ILM has an extremely tight molecular weight (MW) cut-off with 100% sieving at 15 kDa and 0% sieving at 66 kDa. The ability of this new membrane to reject bovine serum albumin (BSA) is shown in Fig. 2A. Its rejection properties (99.6%) are superior to both PSf membrane produced in-house (94.5% rejection) and the published performance of commercial membranes^38,39^. However, despite having the highest BSA rejection, the membrane still had a superior water flux, specifically 21 times greater than PSf and 75–350 times greater than commercial membranes. This finding indicates that the typical performance tradeoff between protein rejection and water flux that is seen in other synthetic polymeric membranes is not absolute^40–42^.

The data on the rejection of blood cell components, including red blood cells, white blood cells, and platelets, demonstrated that the NC-ILM is comparable or superior in each instance to PSf as shown in Table S-2. Molecular weight of uremic toxins vary from 500 to 12,000 Da and, by some estimates, even higher^43^. Using lysozyme as a surrogate for the uremic toxin β2M (beta-2-microglobulin)^44,45^, NC-ILM allowed lysozyme to freely permeate compared to 70% rejection with PSf. The performances of this membrane with anticoagulated (heparin and citrate) porcine blood are shown in Fig. 2B and C, respectively. In both cases the NC-ILM had an initial water flux (evaluated at 40 psig) at least an order of magnitude higher than that of PSf, and after 5–10 h the NC-ILM had maintained a higher water flux than the initial flux of PSf. An experiment was conducted where citrate was removed from the blood originally containing citrate, leaving the blood without an anticoagulant, is shown Fig. 2D. In this case, PSf had zero flux after 2 h and the NC-ILM was still operational after 5 h. Therefore, this new membrane demonstrates greater than a tenfold increase in flux compared to PSf under all operating conditions with blood, and the fouling characteristics are superior to PSf in every case.

**Figure 3:**
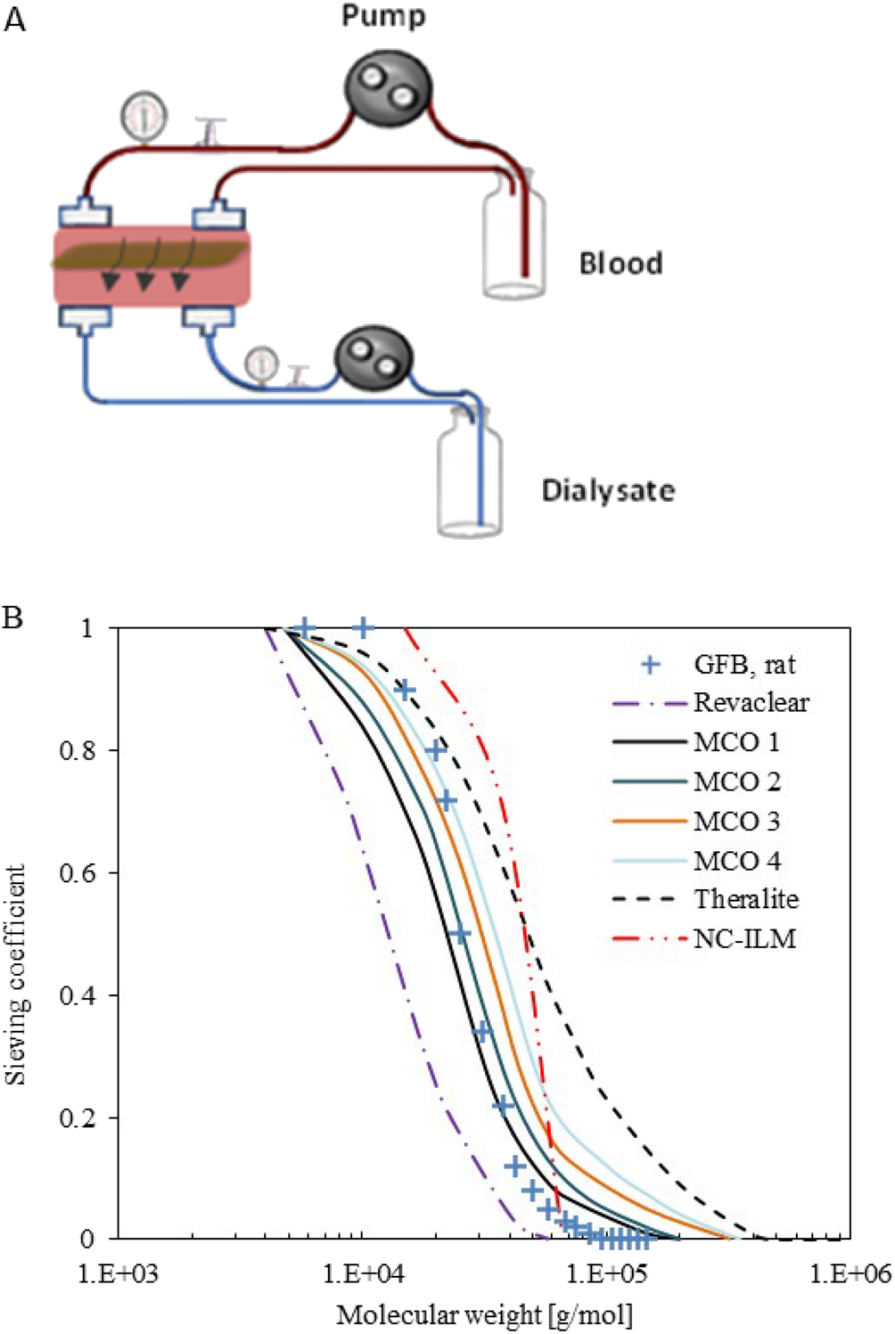
Dialysis performances comparison of different membranes. (**A**) Schematic diagram of the dialysis experimental setup. (**B**) Sieving curves comparison of different membranes showing that the NC-ILM membrane has tightest molecular weight cut-off. (**A**) Generated using Microsoft PowerPoint for Microsoft 365 MSO (Version 2302 Build 16.0.16130.20298) 64-bit using stock images.

Dialysis was performed with this membrane as shown schematically in Fig. 3A. Two different dialysis conditions were tested to determine the appropriate dialysate flow as shown in Table S-3. In these experiments, the dialysis clearance of urea and lysozyme were evaluated. These parameters were selected because removal of urea is of clinical relevance for dialysis patients and lysozyme is a small protein that serves as a surrogate uremic toxin. The urea and middle molecule or surrogate (lysozyme) mass transfer constants are compared in Table 1. The equation and general outline for how these values were calculated are laid out in Supplemental Section S-2. It is interesting to note that the NC-ILM has a urea transport similar to or even better then commercial dialysis membranes^46^ as well as a previously characterized silicone slit membrane^47^. In addition, when comparing middle molecule or surrogate (lysozyme) permeation, the results are even more interesting. The NC-ILM has a much higher mass transfer constant than the silicone slit membrane or commercial dialysis membranes, indicating that uremic toxins with a ~ 14 kDa molecular weight should diffuse through the NC-ILM at a much higher rate. As shown in Fig. 3B, when this data is plotted along with the study of Boschetti-de-Fierro et al.^48^ which used polydisperse dextran as their solutes, the NC-ILM exhibits a very tight MW cut-off as compared to commercial dialysis membranes.

**Table 1:** Mass transfer constants for urea and middle molecular or surrogates in commercial membranes and NC-ILM.

Dialysis unit	Urea Ko (m/s)	Middle molecule or surrogate Ko (m/s)
Optiflux F160NR (Fresenius)	1.30 × 10^–5^	1.03 × 10^–6^ (lysozyme) 8.10 × 10^–7^ (β_2_-microglobulin)
Revaclear (Baxter)	1.39 × 10^–5^	2.64 × 10^–6^ (β_2_-microglobulin)
Silicon Nanopore	2.50 × 10^–6^	4.50 × 10^–7^ (β_2_-microglobulin)
NC-ILM	4.99 × 10^–4^	7.24 × 10^–4^ (lysozyme)

Optiflux urea and lysozyme: https://fmcna.com/content/dam/fmcna/live/products/disposables/dialyzers/additional-resources/Optiflux%20High%20Flux%20Specifications%20Sheet.pdf.

Revaclear urea: https://www.baxter.com/sites/g/files/ebysai746/files/2018-04/Revaclear_Spec_Sheet_FINAL.pdf.

Optiflux and Revaclear β_2_-microglobulin: Misra and Moore, *Hemodialysis International*, 22:S15–S23 (2018).

Silicon nanopore: Kim et al., *PLOS One,* 11:7 (2016).

**Figure 4:**
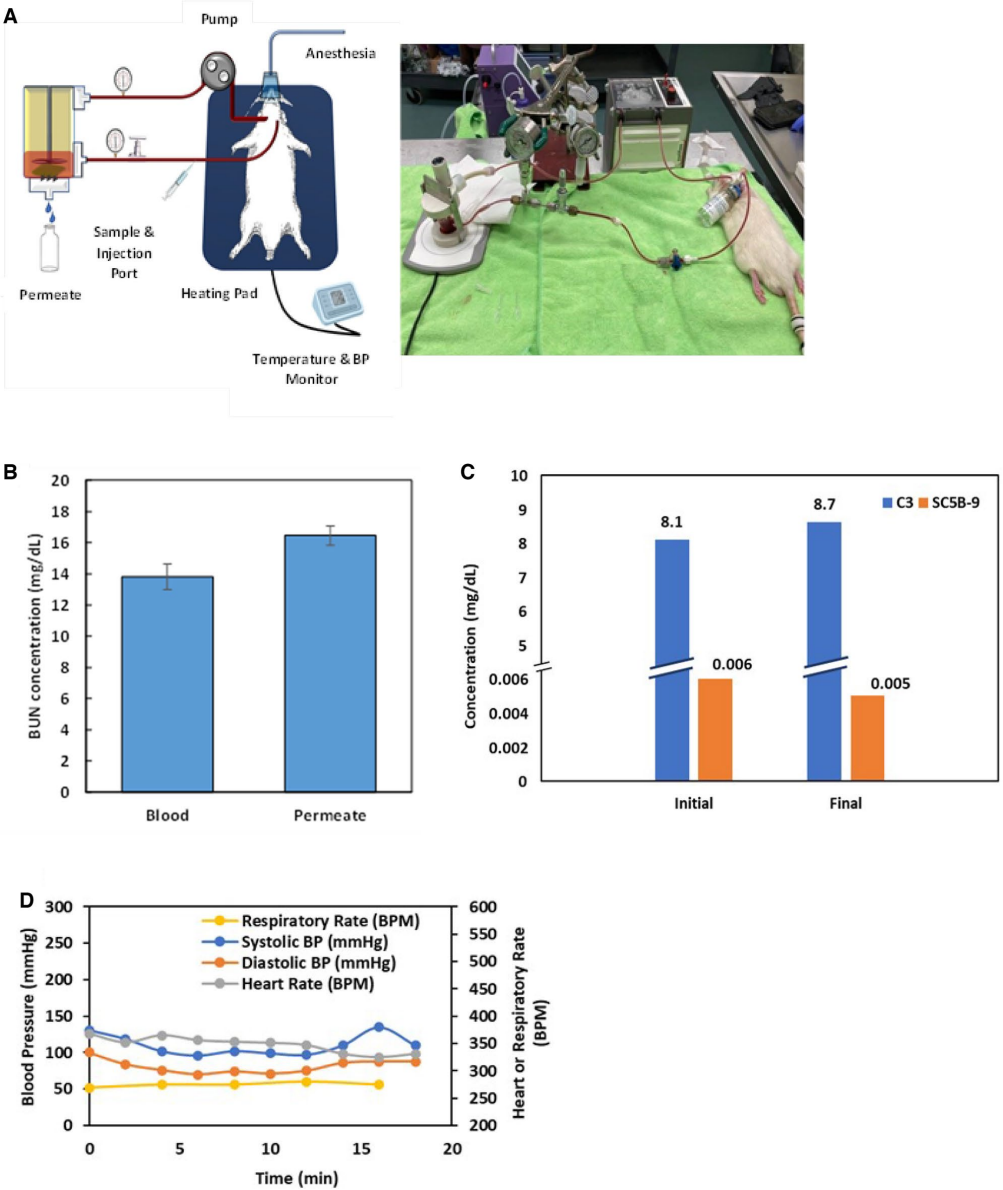
Scheme and results for ex vivo experiments. (**A**) Schematic and picture of ultrafiltration ex vivo experiment. (**B**) Permeation of BUN through the NC-ILM (n = 4). (**C**) Initial and final concentration of complement component C3 (C3) and rat soluble terminal complement complex (SC5B-9) in mg/dL (n = 4). (**D**) Vital measurements from one dialysis experiment (n = 1). Anesthesia level changes and saline administration were provided according to the vitals measurements to keep the animal stable. Both the tubing and membrane casing used in the experiments were made of biocompatible materials. (**A**) Generated using Microsoft PowerPoint for Microsoft 365 MSO (Version 2302 Build 16.0.16130.20298) 64-bit using stock images.

**Figure 5:**
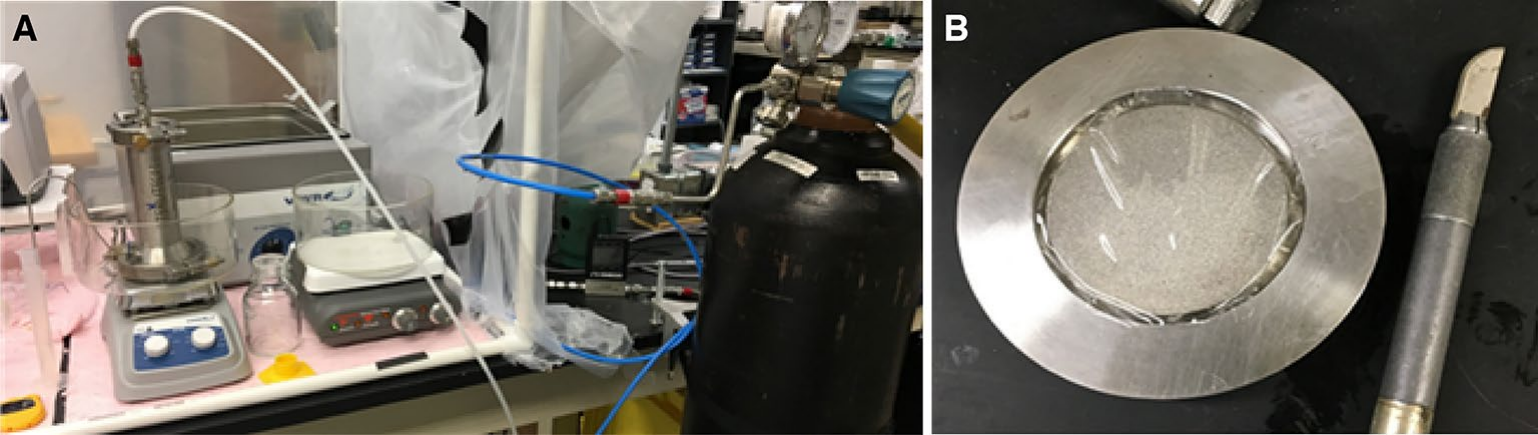
Experimental set-up used for the ultrafiltration experiments. (**A**) Picture of the whole ultrafiltration set-up. (**B**) NC-ILM membrane placement in the stirred cell unit.

To study the ex vivo behavior of the NC-ILM, a rat animal model was developed to test the membrane in both ultrafiltration and dialysis operational modes as shown in Figs. 4A and S-10, respectively. The membrane devices are shown in Figs. S-11 and S-12 for ultrafiltration and in Figs. S-13 and S-14 for dialysis. A schematic of the rat experiment is shown in Fig. 4A. Sprague–Dawley rats were pre-implanted with an access button for femoral vein and artery catheterization, shown in Fig. S-15. The animals were kept under anesthesia during the entire procedure and their vitals were continuously monitored, as shown for a typical animal in Tables S-4 and S-5. Blood from the femoral artery was pumped through the membrane device placed outside the animal’s body and returned to the animal through the femoral vein. The complete flow (single pass) through the extracorporeal circuit took approximately 4 min. The blood urea nitrogen (BUN) concentrations in both the blood and in the permeate are shown in Fig. 4B. The water flux in the ultrafiltration experiments was 7.0 ± 2.1 L/m^2^ h at 3 psig. We also investigated complement activation in rats to evaluate the biocompatibility of the membrane. As shown in Fig. 4C and Table S-6, complement component 3 (C3) and rat soluble terminal complement complex (SC5B-9) did not significantly change before and after permeation, indicating that the NC-ILM is biocompatible in this respect and does not have an appreciable effect on the immune system. In addition, necropsy studies did not reveal any evidence of systemic inflammatory responses induced by the NC-ILM. Indicators of rat wellbeing are shown in Fig. 4D and demonstrate a no significant change during the experiments.

## Discussion

Cellulosic nanomaterials have the potential for high biocompatibility and increased hydrophilicity^49,50^. In this study, cellulose nanofibrils were successfully converted into transparent ultrafiltration membranes comparable to an ionogel. These membranes are oriented in one direction and SEM data shows the appearance of aligned interconnected fibers, an atypical feature of phase inverted polymeric membranes. These interconnected fibers not only have high flux, but also an uncharacteristically sharp molecular weight cut-off not commonly observed in phase inversion membranes. Given the appearance of the interconnected fibers from the SEM data and the sharp molecular weight cut-offs in both ultrafiltration and dialysis mode, one can hypothesize that these novel membranes are behaving as slits rather than cylindrical pores. Kanani et al. performed a study showing that slit membranes do indeed have sharper molecular weight cut-offs than polymeric membranes^37^.

The NC-ILM is made via a phase inversion process and has an unusual structure compared to conventional cellulose. 1-Ethyl-3-methylimidazolium acetate (EMIMAc) is a good solvent for nanocellulose (NC). At 10 wt%, the solutions are viscous, yet well below the solubility limit. During the phase inversion process, water competes with the cellulose phase for EMIMAc, thereby effectively increasing the nanocellulose concentration relative to the ionic liquid within regions of solvated cellulose as shown in the right side of Fig. S-16. Studies involving the phase behavior of EMIMAc-water and EMIMAc-cellulose-water show rapid increases in viscosity within the self-assembled aggregates of the EMIMAc-cellulose, particularly upon the introduction of water prior to coagulation^51^. Although this work was performed with cellulose, it seems reasonable that TEMPO oxidized cellulose will react similarly, albeit with even greater electrostatic interactions due to the highly charged carboxylic groups. It is hypothesized that during the phase inversion process, this self-aggregation kinetically traps a structure like the concentrated state (~ 25 to 70%) observed by Endo et. al^52^. After soaking in excess water, the majority of remaining IL may be exchanged, but an ordered state is retained as seen from SEM (Fig. 1A). The scattering data, as shown in Fig. S-3, corroborates the ordered, IL-mediated self-assembly of cellulose fibers^52^ and is shown schematically in Fig. S-16, adapted from Samayam et al.^53^. Figure 1B shows that the ionic liquid is part of the structure following phase inversion. This could indicate that the cationic imidazolium group electrostatically binds to the C-1 carbon of the cellulosic backbone and surface anion groups that were present in the ILs, which are now exposed to create a negative surface charge^54^. Figure S-9 shows that methanol washing gets rid of evidence of the ionic liquid. However, it should be noted that, even when ionic liquid is no longer detected in the membrane structure, it continues to have the same performance. The ordered structure observed after phase inversion of TEMPO oxidized cellulose was not seen with cellulose and is unique to this material^51–53^.

This novel membrane is proposed as a new dialysis membrane based on its high flux and sharp molecular weight cut-off. Dialysis membranes are divided into various types including low-flux (< 10 mL/min beta-2 microglobulin clearance), high-flux (10–50 mL/min beta-2 microglobulin clearance), and super high-flux (> 50 mL/min beta-2 microglobulin clearance)^55^. These novel NC-ILMs had a urea clearance of 231 mL/min which puts them in the super high-flux category. Recent studies have found that ultra-high flux dialyzers increase survival probability of patients with hemodialysis^55^. However, many ultra-high flux dialyzers also have increased albumin leakage, which can be clinically problematic^56^. Another advantage of the NC-ILMs, as demonstrated in Fig. 2A, is the high flux of urea with little albumin loss. If tunable through changes to the phase inversion process, the tight molecular weight cutoff exhibited by the membranes could be used to control the passage or rejection of low molecular weight uremic toxin, which have been identified as potential contributors to poor patient prognosis, to improve dialysis treatments for patients. In addition, these properties could be useful in a wide range of other industries including protein purification.

## Materials and methods

### Materials

Oxone mediated TEMPO-oxidized cellulose in powder form was synthesized at the University of Arkansas Medical Sciences using a previously published method^57^. Polysulfone pellets (Mw: 75,000) were purchased from Acros Organics (ThermoFisher Scientific, Geel, Belgium). 1-Methyl 2-pyrrolidone (NMP) and 1-Ethyl-3-methylimidazolium acetate (EMIMAc) were acquired from Millipore Sigma (Merck KGaA, Darmstadt, Germany). Bovine serum albumin (BSA) was purchased from VWR USA (Radnor, PA, USA). Heparinized whole porcine blood was purchased from Pel-Freez, LLC (Rogers, AR, USA). All aqueous solutions were prepared using Milli-Q water.

### Membrane casting

A solution of 10 wt% solution Oxone mediated TEMPO-oxidized cellulose was prepared by dissolving the cellulose residues in 1-ethyl-3-methylimidazolium acetate (EMIMAc) ionic liquid. The cast mixture was uniformly mixed on a turning roller for 7 days, ensuring no cellulose lumps were present in the mixture. The same procedure was applied for PSf control membranes as 10 wt% PSf solution was prepared in 1-methyl-2-pyrrolidone (NMP). The mixtures were filtered under vacuum and air bubbles were removed. The homogeneous cast mixtures were then cast onto glass plates via a casting blade, which was adjusted to cast a membrane with a thickness of 200 µm. Cast films were then immersed in a deionized water bath and soaked for 3 min to complete the phase inversion as shown in Fig. S-1.

### Scanning electron microscopy (SEM)

Pictures of the membrane samples were taken in e-SEM mode. The samples were cut into fragments in millimeter size range and sputter-coated (40 mA for 30 s) with an Au/Pd layer in rarefied Argon.

### Fourier transform infrared spectroscopy (FT-IR)

FTIR spectra of the ionic liquid, raw TEMPO oxidized cellulose, and the membrane samples were drawn using Perkin Elmer Frontier FT-IR Spectrometer (PerkinElmer, Waltham, MA, USA). The absorption spectra were taken between wavenumbers 4000 cm^−1^ and 600 cm^−1^ with a scan number of 32 and a resolution of 4 cm^−1^.

### In vitro experiments and sample analyses

To evaluate the filtration performance of the membranes, the experimental set-up shown in Fig. 5A was used. A Sterlitech HP4750 stirred cell was utilized with a membrane area of 20 cm^2^ as shown in Fig. 5B (Sterlitech, Kent, WA, USA). 250 mL of the filtration solution were placed inside the cell chamber, with a magnetic stirrer attached inside, continuously stirring at 200 rpm. The unit was pressurized with N_2_ inert gas, and the pressure was monitored by a digital flow meter. Operating pressure was maintained at 30 psig. Permeate was collected in flasks and flux was manually recorded with a timer. Two filtration media were used for the experiments: a 1 mg/mL bovine serum albumin (BSA) solution for the protein bio-fouling tests and a whole porcine blood solution, anti-coagulated with heparin. BSA (~ 66 kDa), lysozyme (~ 14 KDa) and β-lactoglobulin (~ 37 kDa) contents of the solution samples were measured using a standard bicinchoninic acid (BCA) assay. Complete blood count (CBC) was performed on the blood samples to determine the blood cell concentrations.

### Method for spin-coating and blade-coating for X-ray experiments

Solutions of NC in ILM (5 or 10 wt%) were spin coated on a silicon wafer at 6000 rpm for 60 s. Water was spin coated on top after spin coating the first layer. Blade-coated solutions of NC in ILM (5 or 10 wt%) were cast on a silicon wafer at a gate height of 250 µm. Drops of water were added post blade-coating to induce the phase inversion process.

### Wide-angle X-ray scattering (WAXS) characterization

Grazing-incidence wide-angle X-ray scattering (GIWAXS) was performed at beamline 11^–3^ at the Stanford Synchrotron Radiation Light source (SSRL) at SLAC National Accelerator Laboratory with an X-ray beam energy of 12.7 keV (0.976 Å). The beam defining slits were set to 150 µm (horizontal) × 50 µm (vertical). 2D GIWAXS images were acquired on a Rayonix MX225 CCD area detector comprising 3072 × 3072 pixels with a pixel size of 73.2 × 73.2 µm^2^. The sample to detector distance was set at 300 mm and lanthanum hexaboride (LaB_6_) was used to calibrate the detector orientation. All samples were placed in a helium-filled chamber. Detector images were processed using a combination of pyFAI^58^, pygix, and a custom Python script. The perpendicular scattering was obtained by processing a 15° cake slice offset 5° from the true out-of-plane direction. The parallel scattering was obtained by processing a 15° cake slice offset 5° from the true in-plane direction. X-Ray imaging was performed at beamline 10^–2^ at the Stanford Synchrotron Radiation Light source (SSRL) at SLAC National Accelerator Laboratory. A X-ray beam energy of 9 keV was used on a home-built full-field X-ray camera.

### Ex vivo filtration experiments and sample analysis

The animal studies were conducted in a surgical suite in the Central Laboratory Animal Facility (CLAF), at the University of Arkansas using protocols approved by the IACUC committee and in accordance with relevant guidelines and regulations. The methods are reported in accordance with ARRIVE guidelines. Sprague–Dawley male rats pre-implanted with femoral vein and artery catheters were ordered from Envigo (MA, USA). All animals were kept under anesthesia (isoflurane via nose cone at 2–4% initially and then maintained at 1–1.5%) during the whole filtration process. CODA tail-cuff monitoring device (CT, USA, purchased from Kent Scientific Corporation) was used to continuously measure their vitals such as blood pressure, heart rate, and temperature. Initially, the animal was slowly heparinized with 0.5 mL of the 100 IU/mL heparin on each port adapter. Once the vitals were considered stable, the extra corporeal circuit was connected to the animal through the arterial port. Then the dialysate (if dialysis experiment) and blood pumps were initiated at approximately 10 mL/min and 1 mL/min, respectively. After 2 or 3 drops of saline at the end of the circuit, the femoral port was connected to the animal as shown in Fig. S-15. For ultrafiltration experiments, a pressure of 2–3 psi was applied on the membrane. Anesthetic levels or saline administration were adjusted based on the vitals measurements. At the end of each experiment, euthanasia (using 5% isoflurane followed by bilateral pneumothorax) was performed before the animal woke up.

Enzyme-linked Immunosorbent Assay Kit for Complement Component 3 (C3) and Rat Soluble Terminal Complement Complex (SC5B-9) ELISA Kit were used to test the complement activation. In order to improve the biocompatibility of the device the casing was 3-D printed using “Biomed Clear Resin” by Formlabs (Boston, MA), which was specifically designed for biocompatible applications requiring long-term skin or mucosal membrane contact. In addition the tubing was from the Pharmed BPT biocompatible tubing series (Saint-Gobain US, Malvern, PA), which is commonly used for biopharmaceutical applications.

## Supplementary Information

Supplementary Information.

Supplementary Figures.

## Data Availability

The datasets used and/or analyzed during the current study may be made available by the corresponding author upon reasonable request.
