# Supplementary material for: High flux novel polymeric membrane for renal applications
Source: Sci Rep. 2023 Jul 20;13:11703. doi: 10.1038/s41598-023-37765-y (PMC10359412; doi:10.1038/s41598-023-37765-y)
Supplement: Supplementary file 1 — Supplementary Information. [file 41598_2023_37765_MOESM1_ESM.docx]

**Supplemental Section S-1**

Average pore radius and porosity were calculated using the Guerout–Elford–Ferry method^1^. Briefly, porosity of the membrane samples were calculating using a gravimetric method, with the equation given below:

|  | $\varepsilon=\frac{(\frac{w_{1}-w_{2}}{D_{w}})}{(\frac{w_{1}-w_{2}}{D_{w}})+\frac{w_{2}}{D_{p}}}$ | Equation 1 |
| --- | --- | --- |

where *w_1_* and *w_2_* are the wet and dry weight of the membrane sample, and *D_w_* and *D_p_* are the density of water and the polymer, respectively. Calculated porosity values were then placed in the following equation:

|  | $R_{f}= \sqrt{\frac{\left( 2.90-1.75\varepsilon\right)8\eta hQ}{\varepsilon PA}}$ | Equation 2 |
| --- | --- | --- |

where η is water viscosity, *h* is the thickness of the membrane coupon, *Q* is the permeated water flux per time, *P* is the operating pressure and *A* is the membrane area. The calculation for a single NC-ILM membrane coupon using Equation 1 is shown in Table S-7. Similarly, average pore radius was calculated using Equation 2 and is shown in Table S-8. Samples were analyzed in triplicate.

Table S-7: Example of pore radius calculation (ε) values

| ε (calculated) | Wet membrane weight (mg) | Dry membrane weight (mg) | Water density (g/cm^3^) | Polymer density (g/cm^3^) |
| --- | --- | --- | --- | --- |
| **0.94** | 50.55 | 3.74 | 0.993 | 1.21 |

Table S-8: Example calculation of average pore radius (R_f_) values

| R_f_, Average pore radius (nm) | Pure water viscosity (mPa.s) | Membrane thickness (µm) | Permeate flowrate (mL/min) | Pressure (MPa) | Membrane area (m^2^) |
| --- | --- | --- | --- | --- | --- |
| **13.6** | 0.8891 | 200 | 0.2625 | 0.206 | 0.0002 |

**Reference**

1 Feng, C., Shi, B., Li, G. & Wu, Y. Preparation and properties of microporous membrane from poly(vinylidene fluoride-co-tetrafluoroethylene) (F2.4) for membrane distillation. *Journal of Membrane Science* **237**, 15-24 (2004). <https://doi.org:10.1016/S0376-7388(04)00107-3>|10.1016/j.memsci.2004.02.007

**Supplemental Section S-2**

The *K_o_A* (mass transfer area constant) equation for solid clearance from the blood is^2^:

$K_{o}A=\frac{Q_{B}*Q_{D}}{Q_{B}-Q_{D}}\ln\left[ \frac{1-{(K_{D}/Q}_{B})}{1-{(K_{D}/Q}_{D})} \right]$ Equation 3

where K_D_ is the solute clearance, Q_B_ and Q_D_ are the chosen blood and dialysate flow rates, respectively. The experimental urea clearance (K_D_) was calculated for the given specific solute to be transported through the membrane, by the equation given below^3^:

$K_{D}=\frac{C_{in}-C_{out}}{C_{in}}* Q_{B}$ Equation 4

where C_in_ and C_out_ are initial and final solute blood concentrations, respectively. Urea clearance and mass transfer area constant values were determined by measuring the initial concentration and the final concentration over a four-hour experiment. From the experimental data, urea clearance values and *KoA* for a single pool dialysis run were calculated as given in Table S-9 To be able to compare the performance of the NC-ILM to commercial dialyzers, the mass transfer constant (K_o_) was calculated using Equation 3 and dividing by the surface area of the unit. Calculations using the same process were performed for lysozyme which was used as a surrogate for uremic toxins such as middle molecules.

Table S-9: Calculated urea clearances and mass transfer constants

| **Blood flow rate (mL/min)** | **Dialysate flow rate (mL/min)** | **Experimental urea clearance (mL/min)** | ***KoA* (mL/min)** | ***Ko* (m/s)** |
| --- | --- | --- | --- | --- |
| 100 | 138 | 26.26 | 33.95 | 4.99*10^-4^ |

The *in vitro* ultrafiltration constant (K_UF_) of the dialyzer is calculated with the equation given below^4^:

$K_{UF}= \frac{Q_{UF}}{TMP*A}$ and $\mathrm{TMP}= \frac{P_{inlet}+P_{outlet}}{2}$ Equation 5

where, Q_UF_ is the ultrafiltration rate of water for a closed-loop dialysis operation. Typically, the unit of measurement for K_UF_ is mL/h/mmHg/m^2^ and is used to classify dialyzers as low or high flux in terms of water permeability^5^.

Finally, the sieving coefficient (S), which is generally used to characterize the convective transport properties of a given solute, was calculated for the membranes by the equation listed below^5^:

$S= \frac{C_{F}}{C_{P}}$ Equation 6

where, C_F_ and C_P_ are feed and permeate concentrations of bovine serum albumin (BSA), the model solute used for the experiments to observe the effects of the secondary membrane formed on the dialyzer surface. Theoretical sieving coefficients were determined via the sieving model calculations given by Kim et al^6^. Briefly a series of calculations based on the hydrodynamic pore models were conducted for three solutes (proteins with three different MWs) and the optimized fit values were reported by the minimization of the sum of the squared residuals between the experimental data and the model outputs.

**References**

1 Feng, C., Shi, B., Li, G. & Wu, Y. Preparation and properties of microporous membrane from poly(vinylidene fluoride-co-tetrafluoroethylene) (F2.4) for membrane distillation. *Journal of Membrane Science* **237**, 15-24 (2004). <https://doi.org:10.1016/S0376-7388(04)00107-3>|10.1016/j.memsci.2004.02.007

2 Leypoldt, J. K. *et al.* Hemodialyzer mass transfer-area coefficients for urea increase at high dialysate flow rates. The Hemodialysis (HEMO) Study. *Kidney Int* **51**, 2013-2017 (1997). <https://doi.org:10.1038/ki.1997.274>

3 Daugirdas, J. T., Blake, P. G. & Ing, T. S. *Handbook of dialysis*. Fifth edition. edn, (Wolters Kluwer Health, 2015).

4 Ficheux, A., Ronco, C., Brunet, P. & Argilés, À. The ultrafiltration coefficient: this old 'grand inconnu' in dialysis. *Nephrol Dial Transplant* **30**, 204-208 (2015). <https://doi.org:10.1093/ndt/gft493>

5 Ronco, C. & Clark, W. R. Haemodialysis membranes. *Nat Rev Nephrol* **14**, 394-410 (2018). <https://doi.org:10.1038/s41581-018-0002-x>

6 Kim, T. R., Hadidi, M., Motevalian, S. P., Sunohara, T. & Zydney, A. L. Transport Characteristics of Asymmetric Cellulose Triacetate Hemodialysis Membranes. *Blood Purif* **45**, 46-52 (2018). <https://doi.org:10.1159/000480491>
