# Supplementary material for: High flux novel polymeric membrane for renal applications
Source: Sci Rep. 2023 Jul 20;13:11703. doi: 10.1038/s41598-023-37765-y (PMC10359412; doi:10.1038/s41598-023-37765-y)
Supplement: Supplementary file 2 — Supplementary Figures. [file 41598_2023_37765_MOESM2_ESM.pptx]

## Slide 1
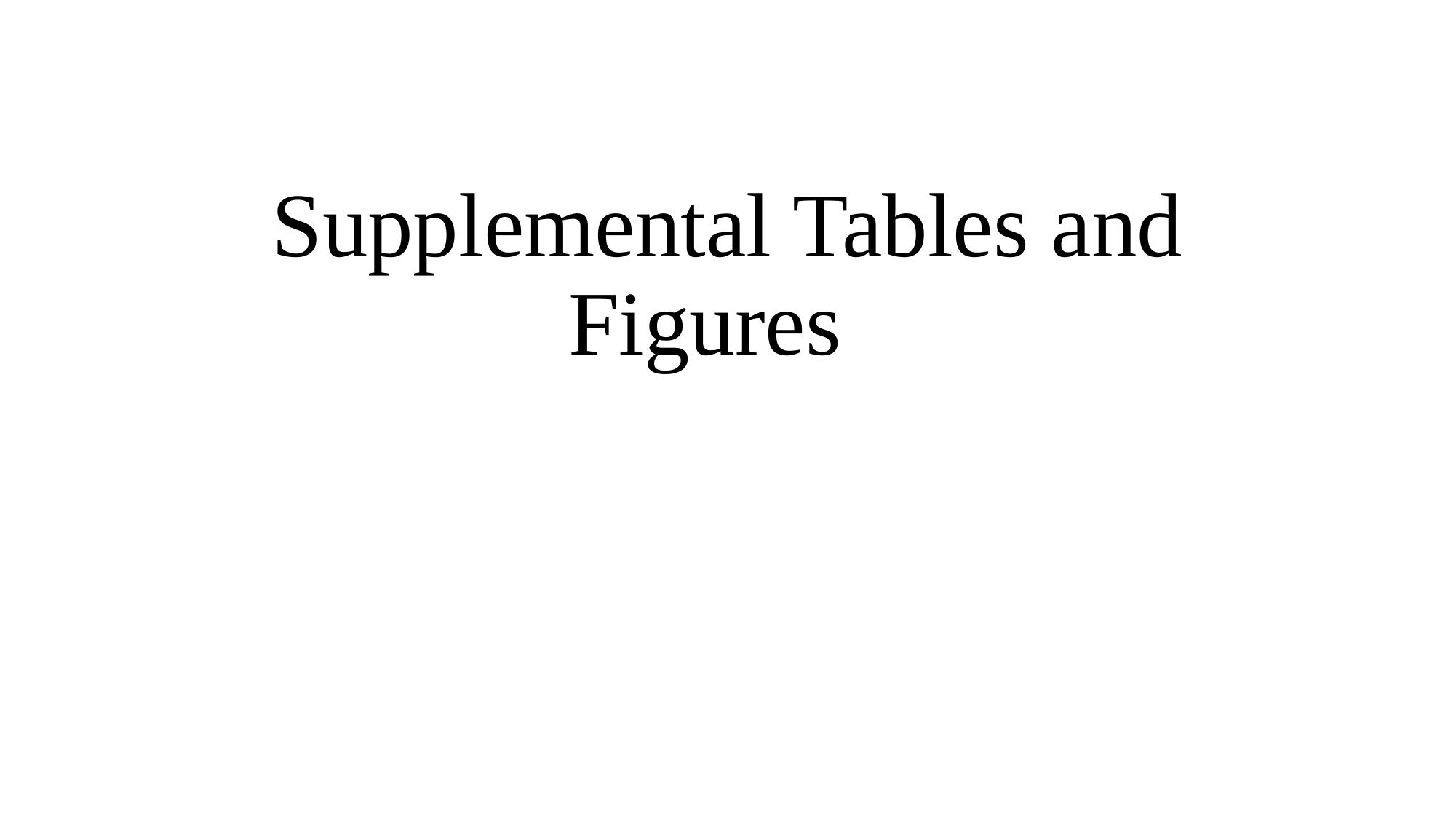

# Supplemental Tables and Figures

## Slide 2
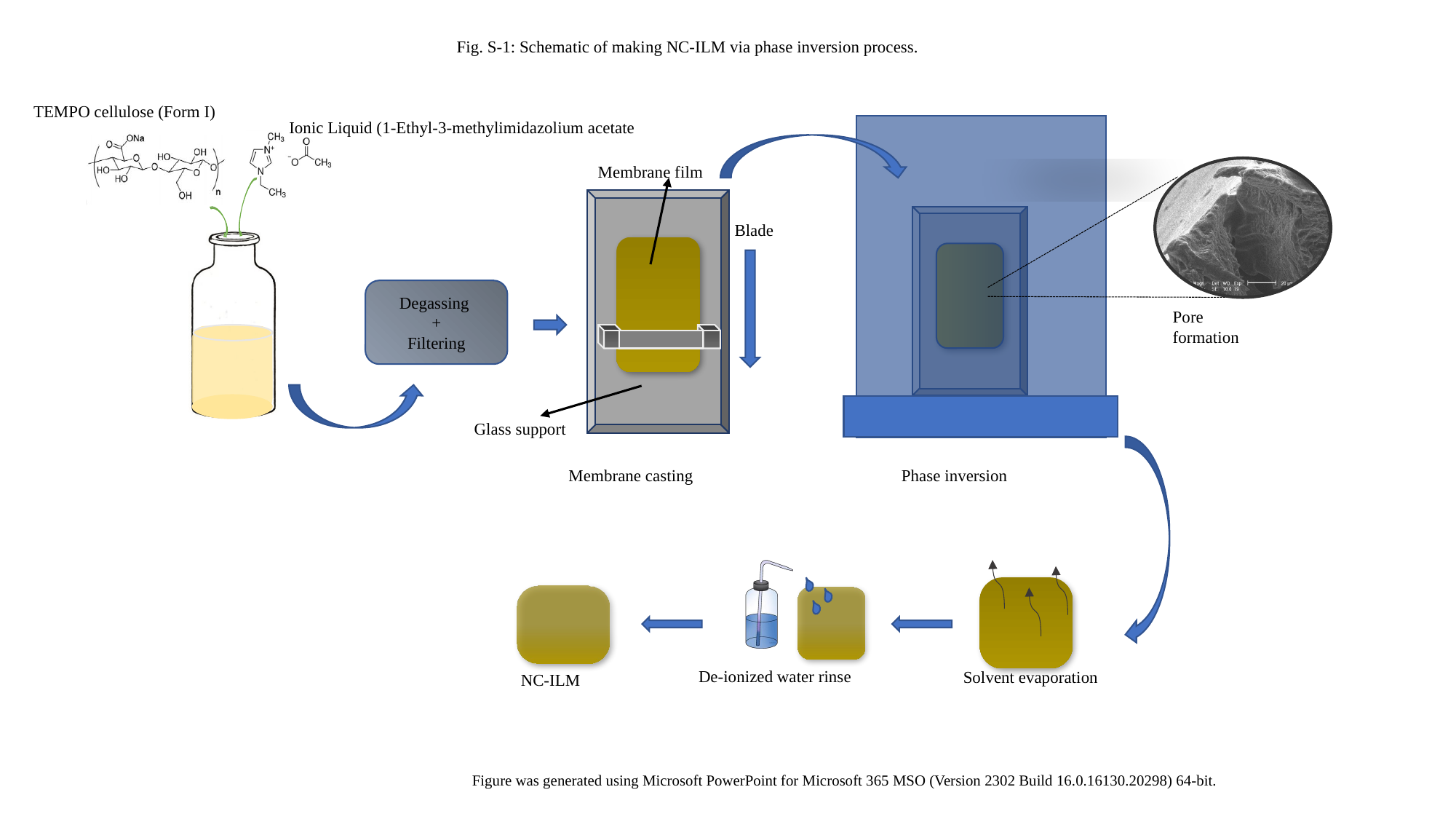

# Fig. S-1: Schematic of making NC-ILM via phase inversion process.
TEMPO cellulose (Form I)
Ionic Liquid (1-Ethyl-3-methylimidazolium acetate
Membrane film
Blade
Pore formation
Glass support
Membrane casting
Phase inversion
De-ionized water rinse
Solvent evaporation
NC-ILM
Degassing
+
Filtering
Figure was generated using Microsoft PowerPoint for Microsoft 365 MSO (Version 2302 Build 16.0.16130.20298) 64-bit.

## Slide 3
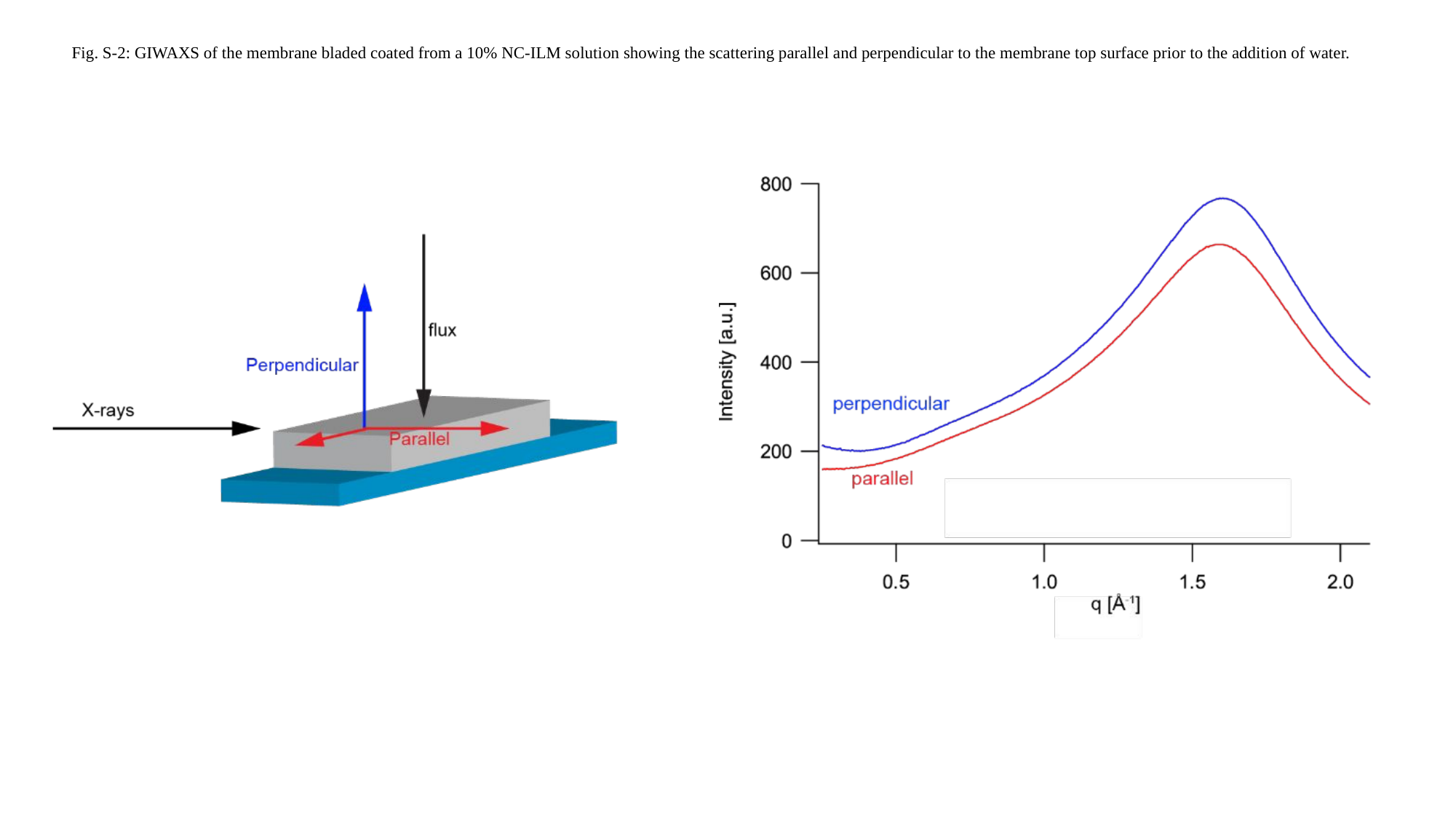

Fig. S-2: GIWAXS of the membrane bladed coated from a 10% NC-ILM solution showing the scattering parallel and perpendicular to the membrane top surface prior to the addition of water.

## Slide 4
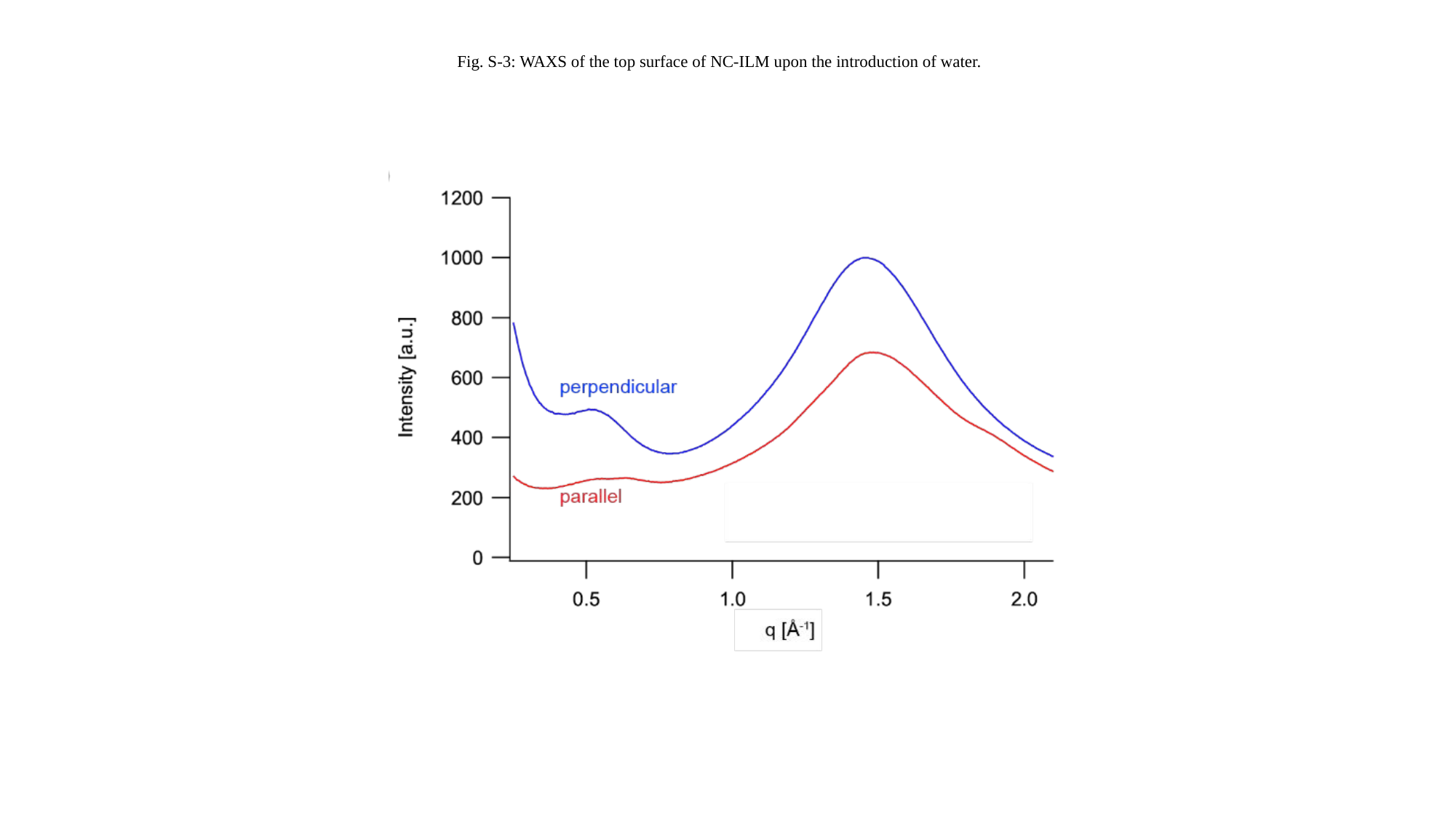

Fig. S-3: WAXS of the top surface of NC-ILM upon the introduction of water.

## Slide 5
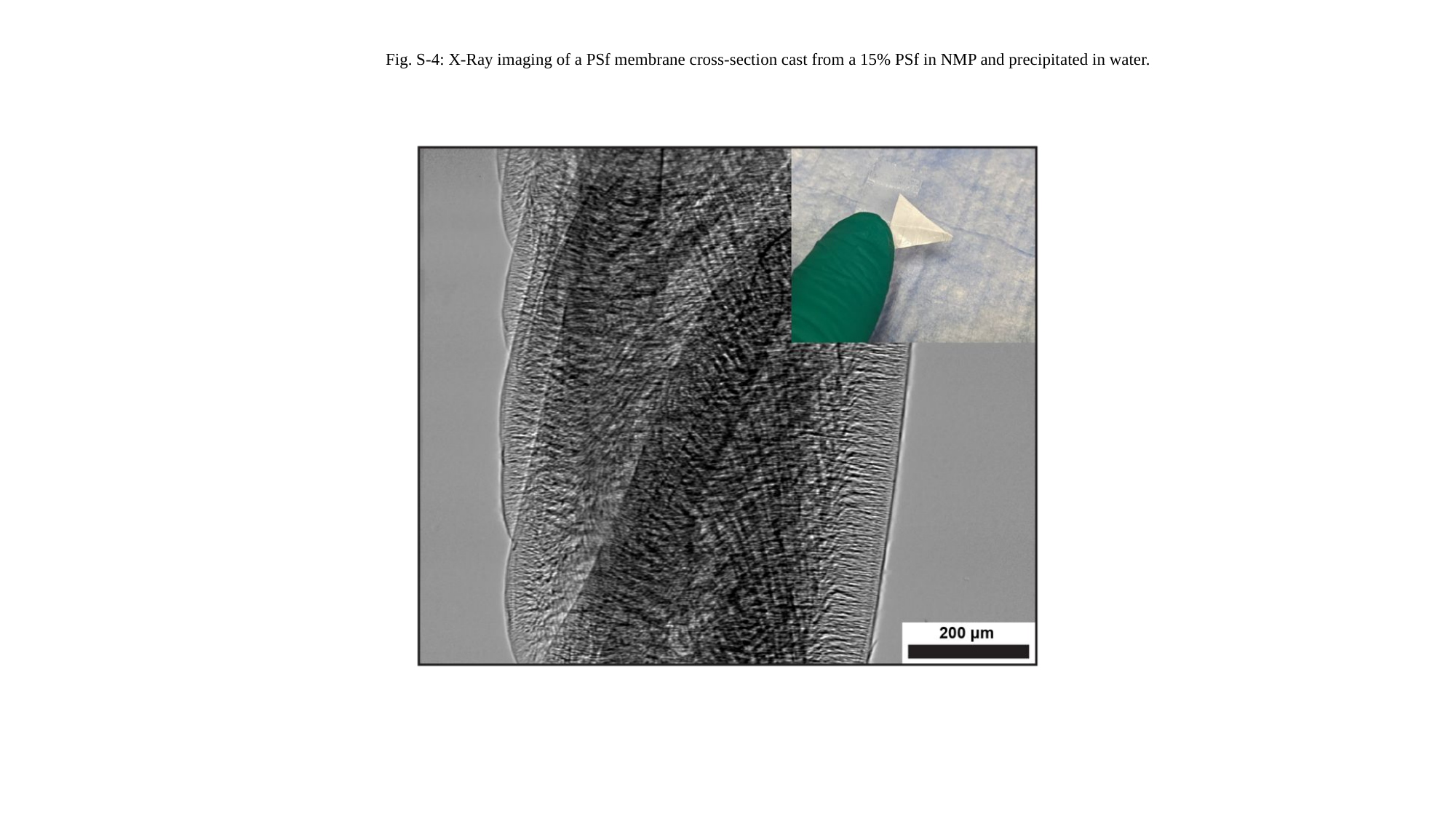

Fig. S-4: X-Ray imaging of a PSf membrane cross-section cast from a 15% PSf in NMP and precipitated in water.

## Slide 6
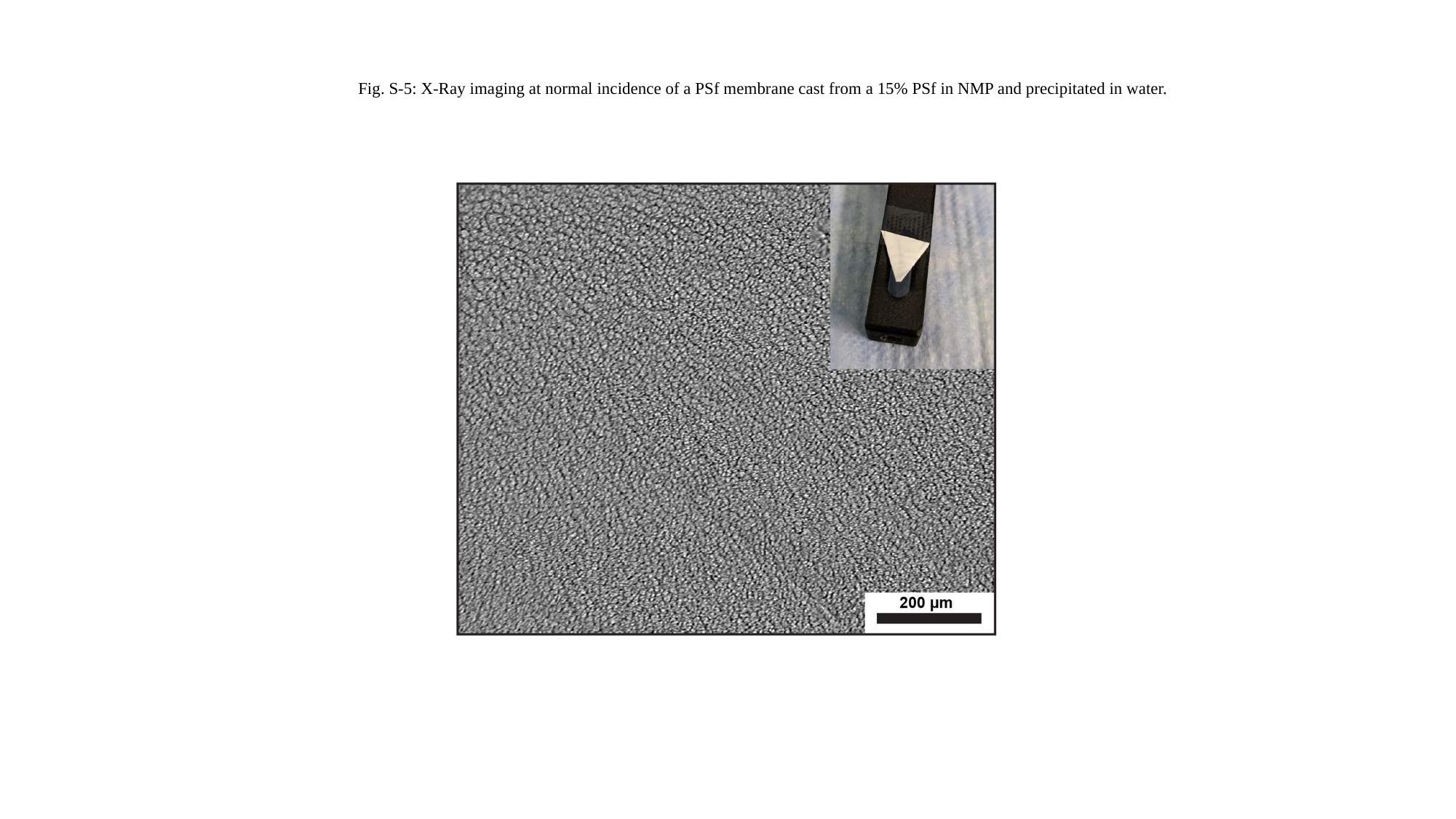

Fig. S-5: X-Ray imaging at normal incidence of a PSf membrane cast from a 15% PSf in NMP and precipitated in water.

## Slide 7
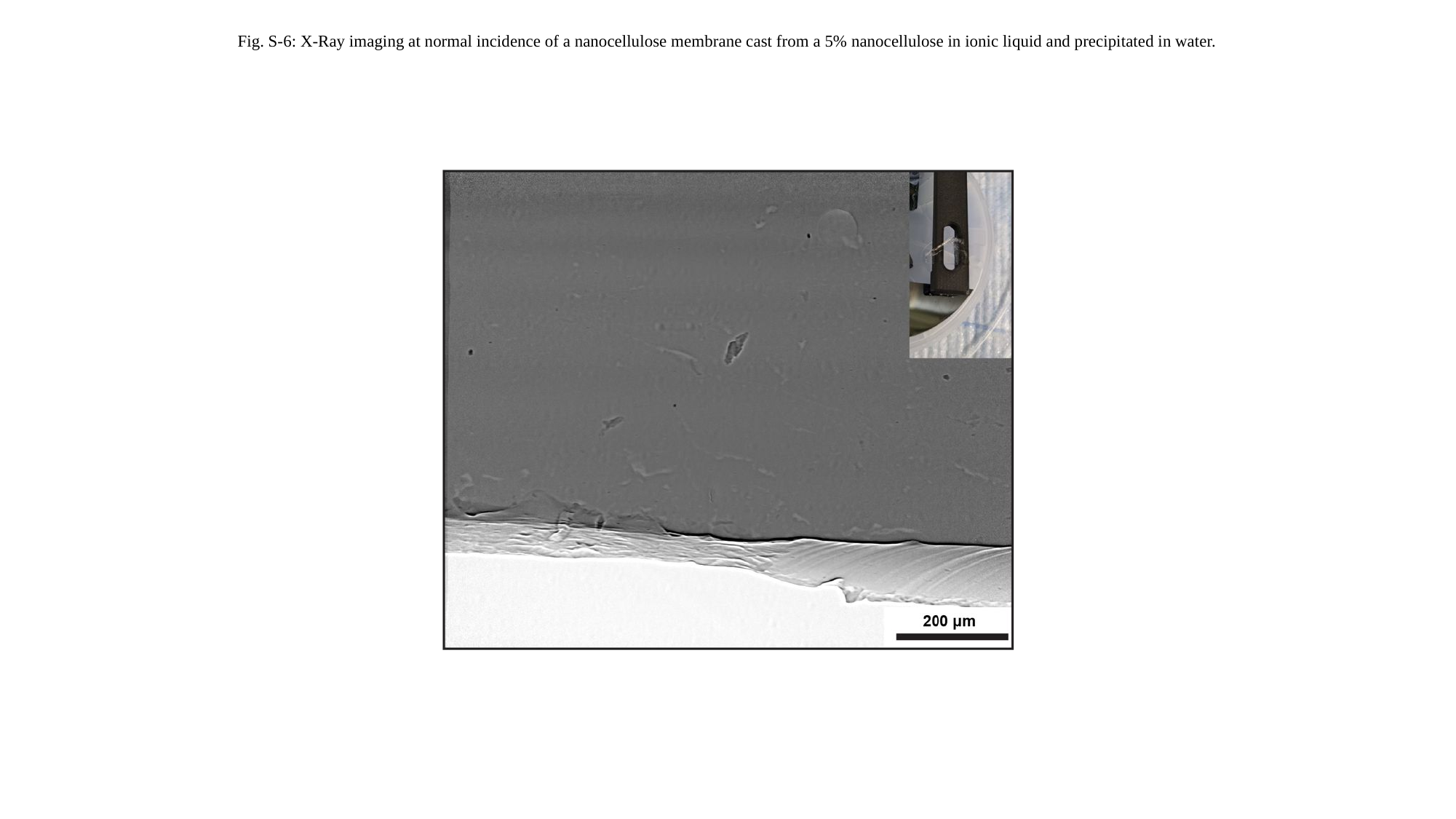

Fig. S-6: X-Ray imaging at normal incidence of a nanocellulose membrane cast from a 5% nanocellulose in ionic liquid and precipitated in water.

## Slide 8
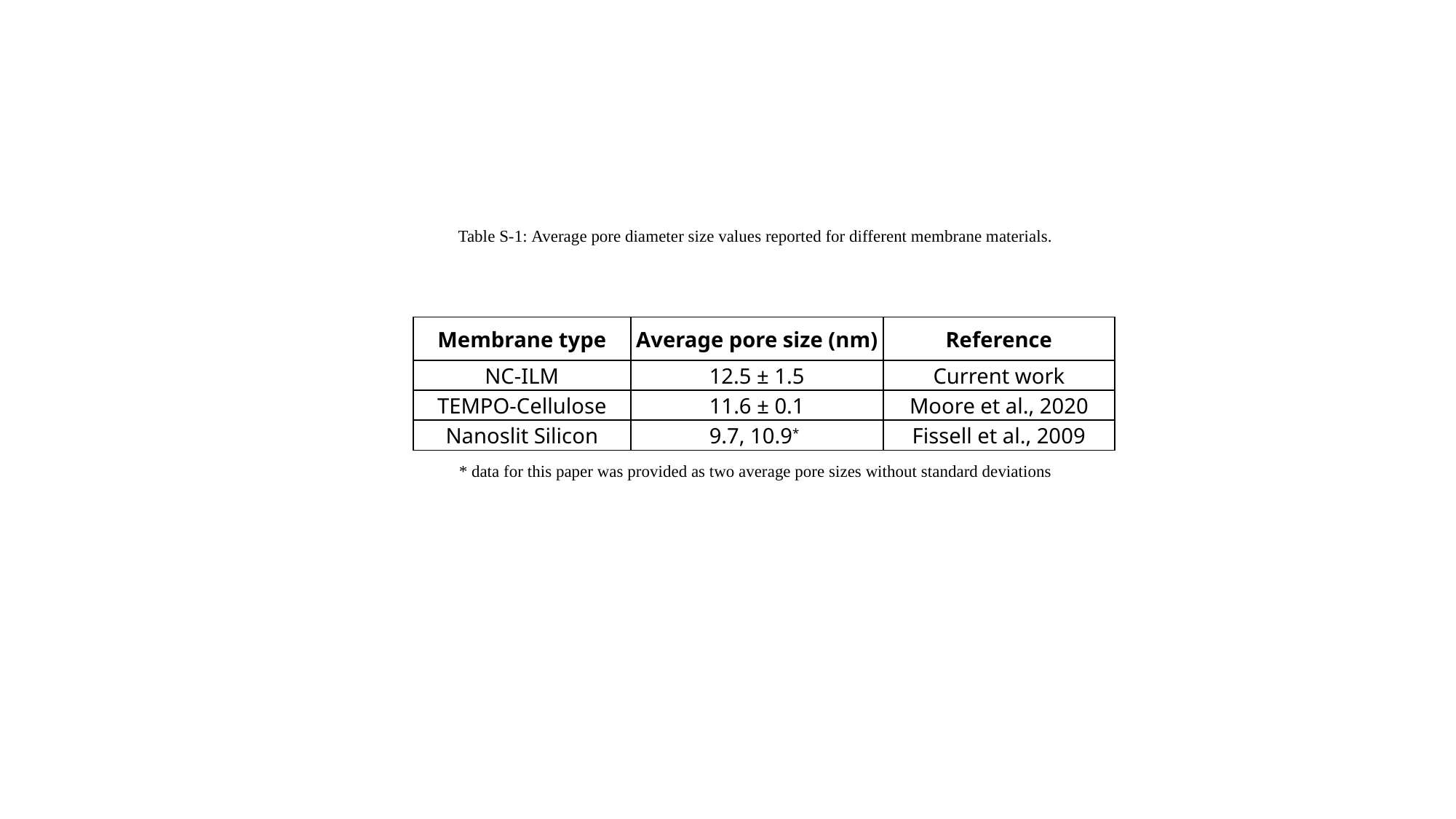

Table S-1: Average pore diameter size values reported for different membrane materials.
| Membrane type | Average pore size (nm) | Reference |
| --- | --- | --- |
| NC-ILM | 12.5 ± 1.5 | Current work |
| TEMPO-Cellulose | 11.6 ± 0.1 | Moore et al., 2020 |
| Nanoslit Silicon | 9.7, 10.9\* | Fissell et al., 2009 |
* data for this paper was provided as two average pore sizes without standard deviations

## Slide 9
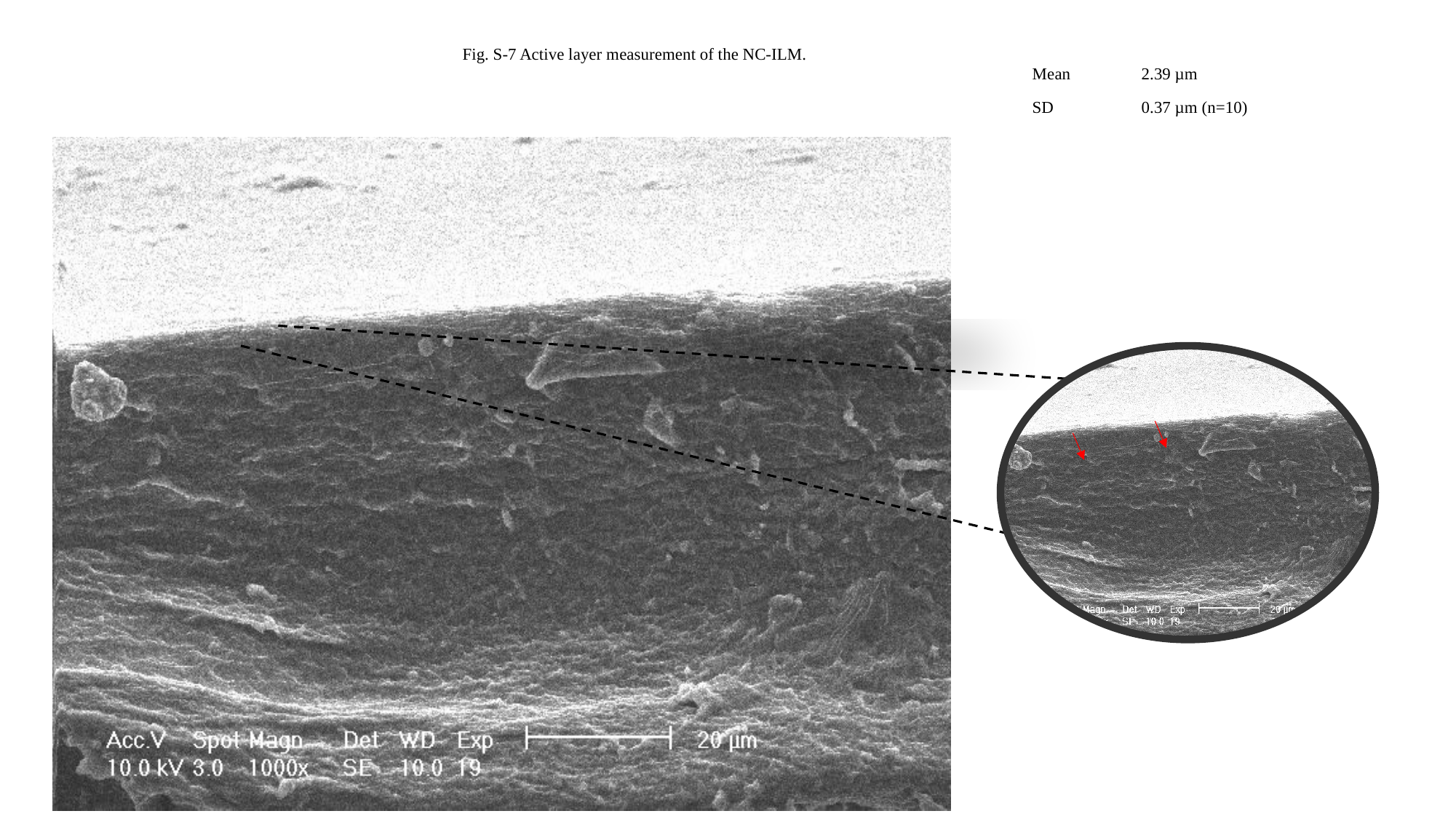

Fig. S-7 Active layer measurement of the NC-ILM.
Mean	2.39 µm
SD	0.37 µm (n=10)

## Slide 10
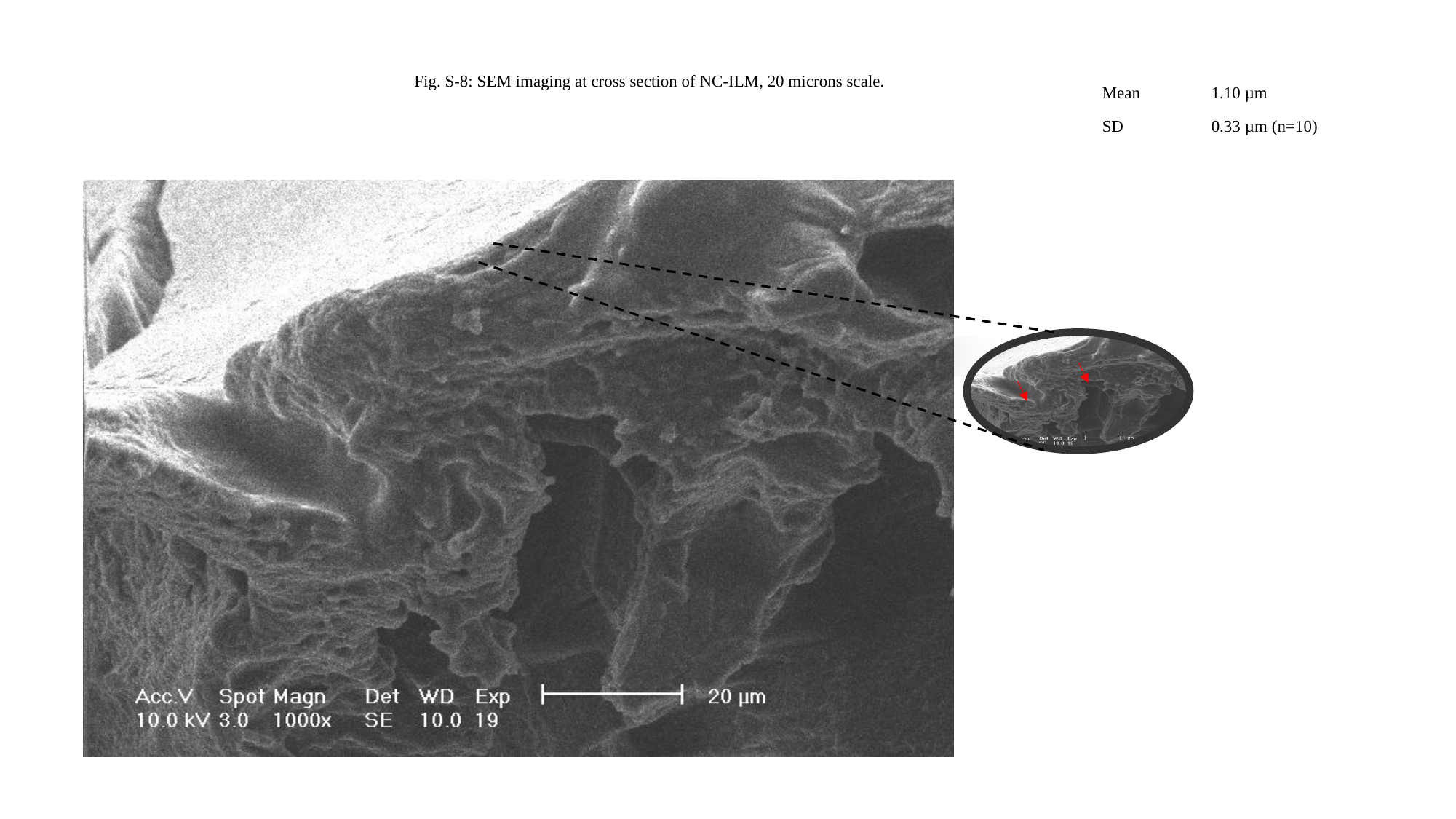

Fig. S-8: SEM imaging at cross section of NC-ILM, 20 microns scale.
Mean	1.10 µm
SD	0.33 µm (n=10)

## Slide 11
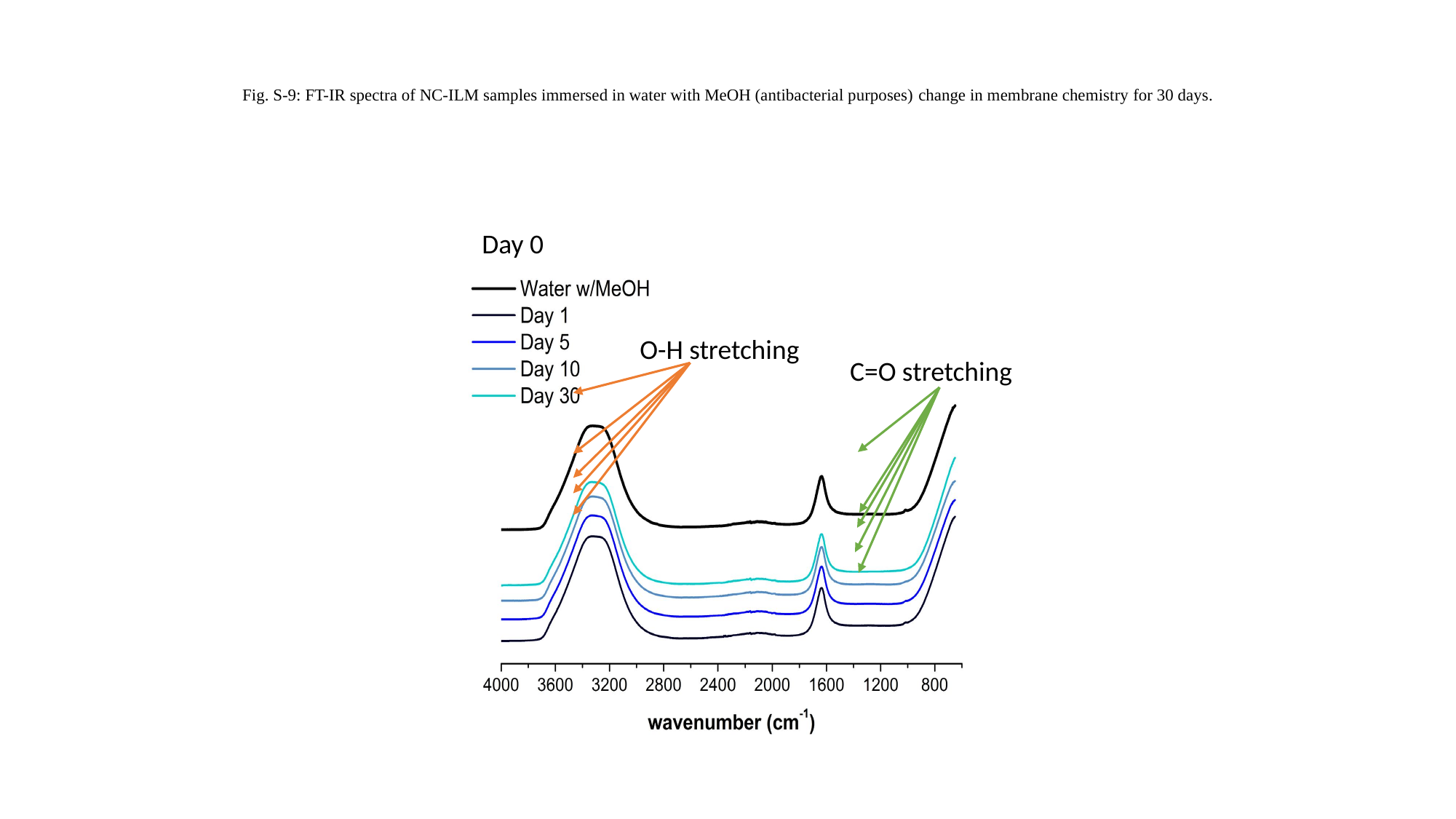

# Fig. S-9: FT-IR spectra of NC-ILM samples immersed in water with MeOH (antibacterial purposes) change in membrane chemistry for 30 days.
Day 0
O-H stretching
C=O stretching

## Slide 12
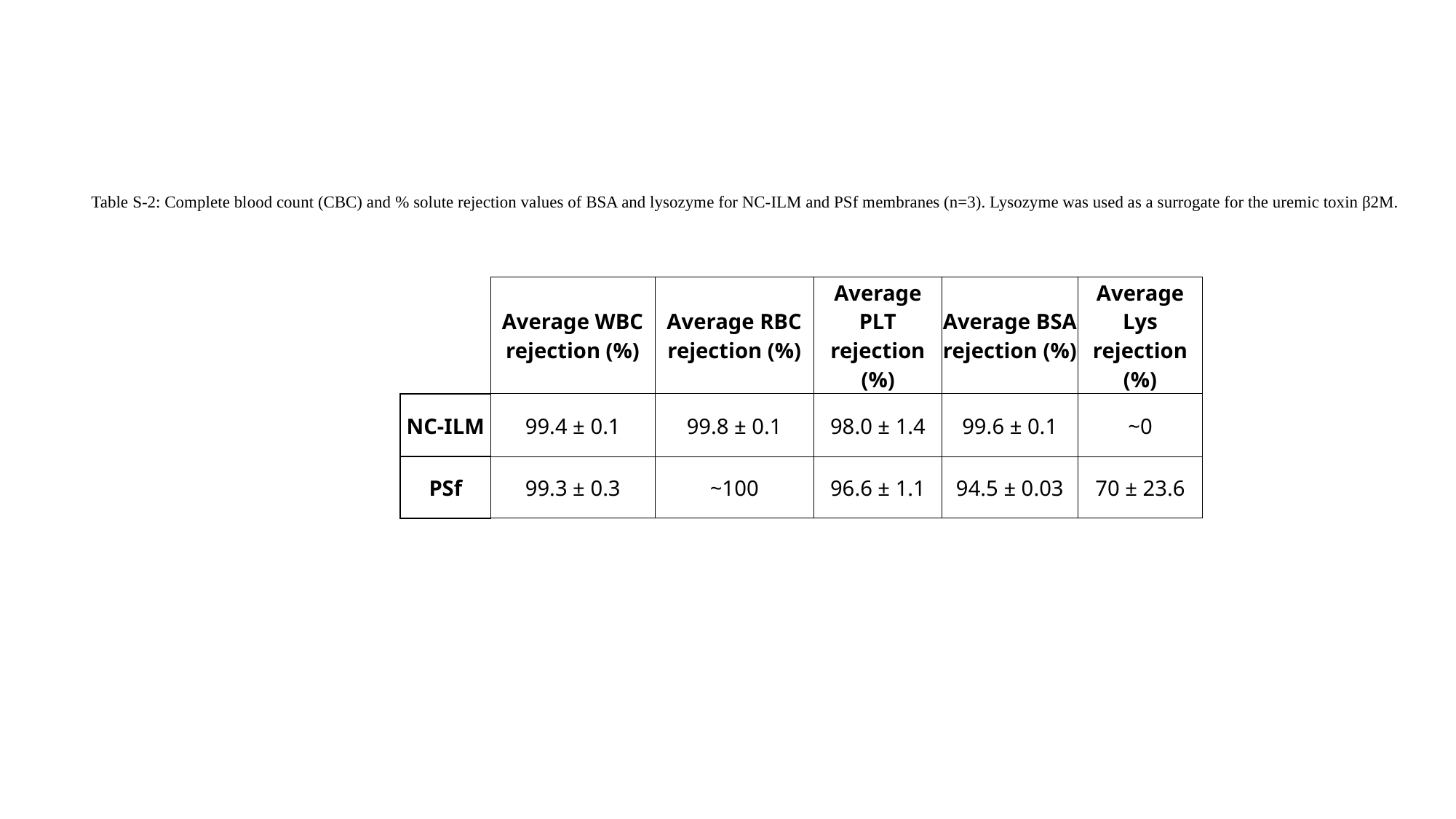

Table S-2: Complete blood count (CBC) and % solute rejection values of BSA and lysozyme for NC-ILM and PSf membranes (n=3). Lysozyme was used as a surrogate for the uremic toxin β2M.
| | Average WBC rejection (%) | Average RBC rejection (%) | Average PLT rejection (%) | Average BSA rejection (%) | Average Lys rejection (%) |
| --- | --- | --- | --- | --- | --- |
| NC-ILM | 99.4 ± 0.1 | 99.8 ± 0.1 | 98.0 ± 1.4 | 99.6 ± 0.1 | ~0 |
| PSf | 99.3 ± 0.3 | ~100 | 96.6 ± 1.1 | 94.5 ± 0.03 | 70 ± 23.6 |

## Slide 13
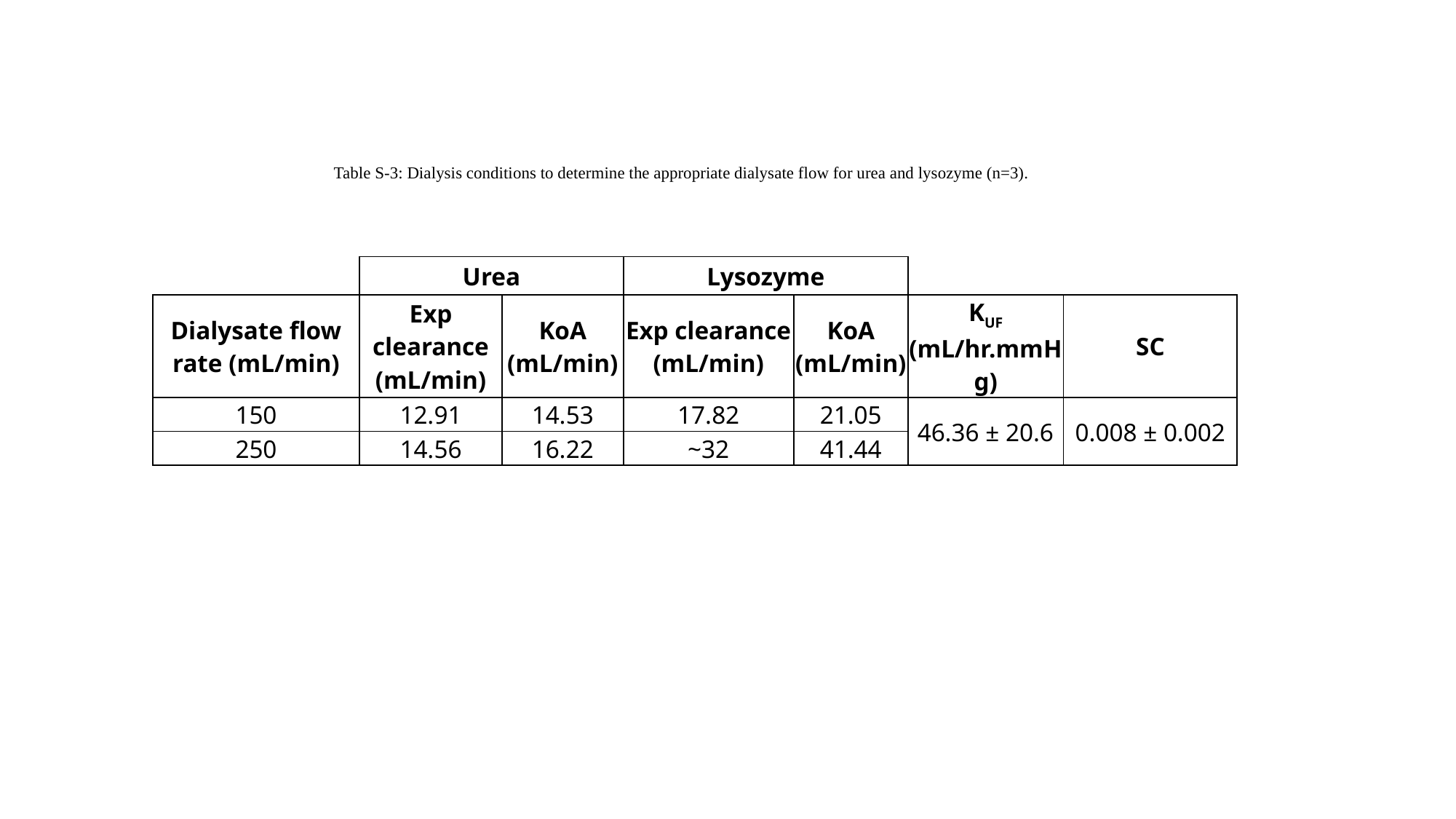

Table S-3: Dialysis conditions to determine the appropriate dialysate flow for urea and lysozyme (n=3).
| | Urea | | Lysozyme | | | |
| --- | --- | --- | --- | --- | --- | --- |
| Dialysate flow rate (mL/min) | Exp clearance (mL/min) | KoA (mL/min) | Exp clearance (mL/min) | KoA (mL/min) | KUF (mL/hr.mmHg) | SC |
| 150 | 12.91 | 14.53 | 17.82 | 21.05 | 46.36 ± 20.6 | 0.008 ± 0.002 |
| 250 | 14.56 | 16.22 | ~32 | 41.44 | | |

## Slide 14
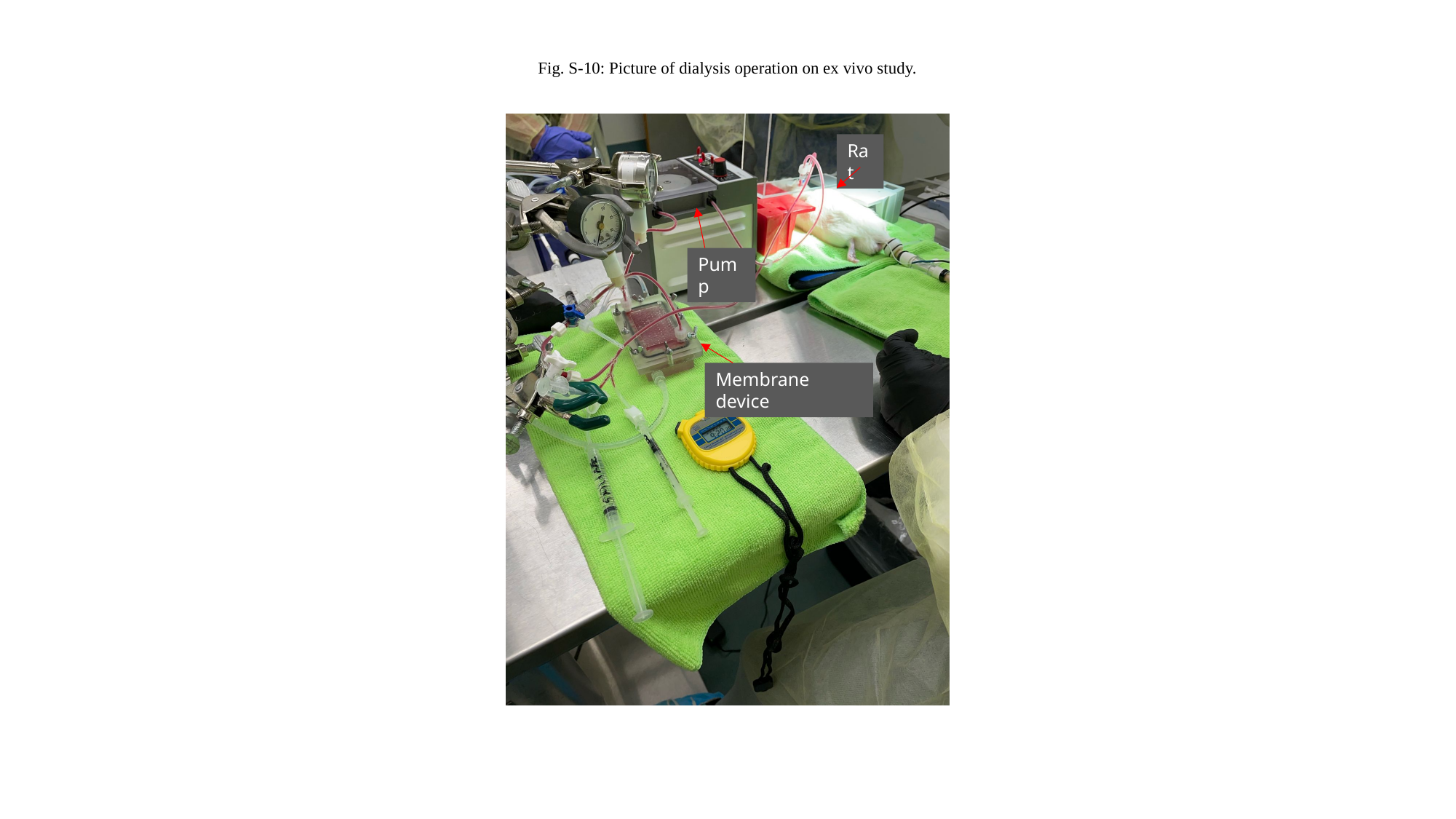

Fig. S-10: Picture of dialysis operation on ex vivo study.
Rat
Pump
Membrane device

## Slide 15
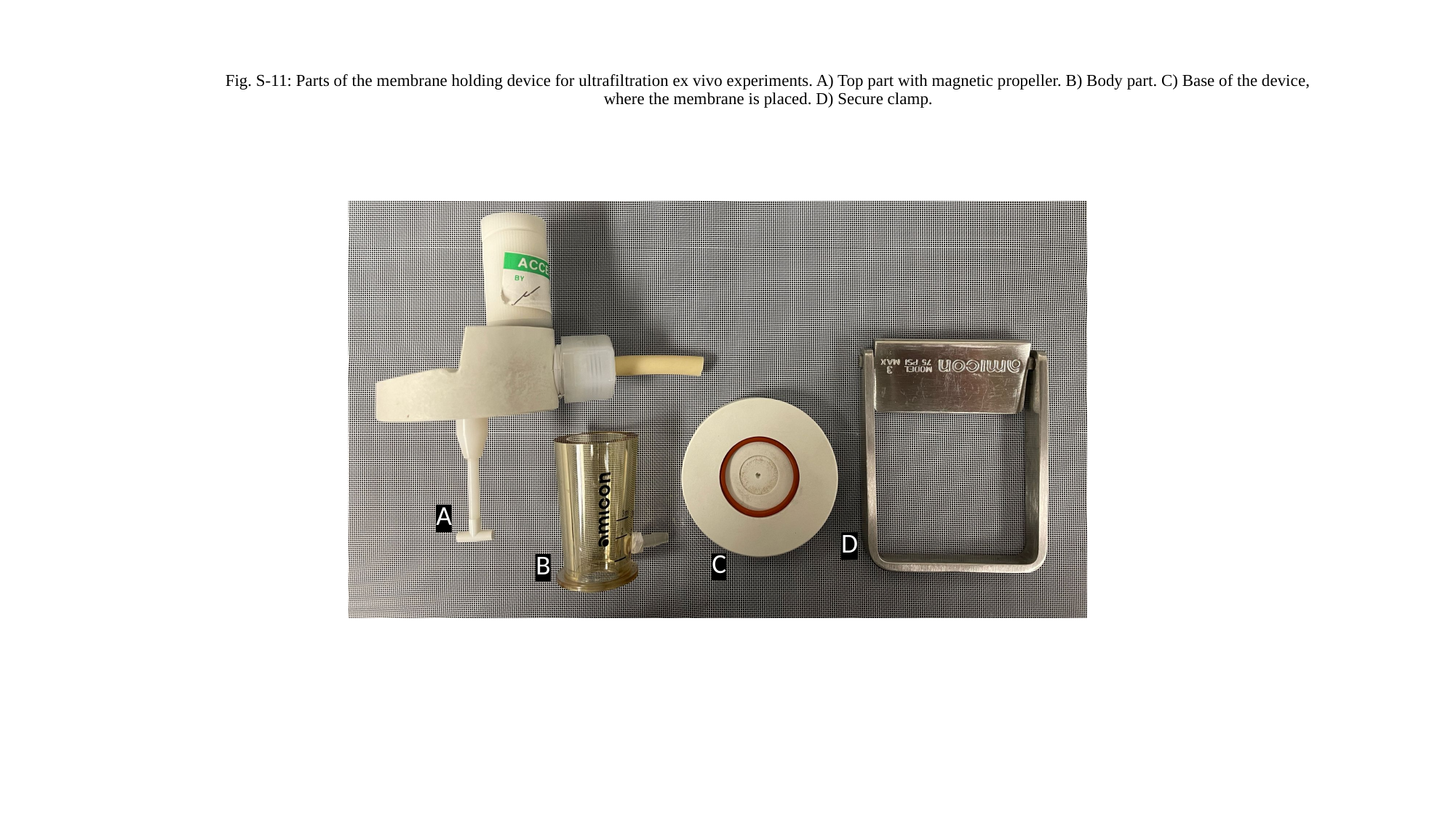

Fig. S-11: Parts of the membrane holding device for ultrafiltration ex vivo experiments. A) Top part with magnetic propeller. B) Body part. C) Base of the device, where the membrane is placed. D) Secure clamp.
A
D
C
B

## Slide 16
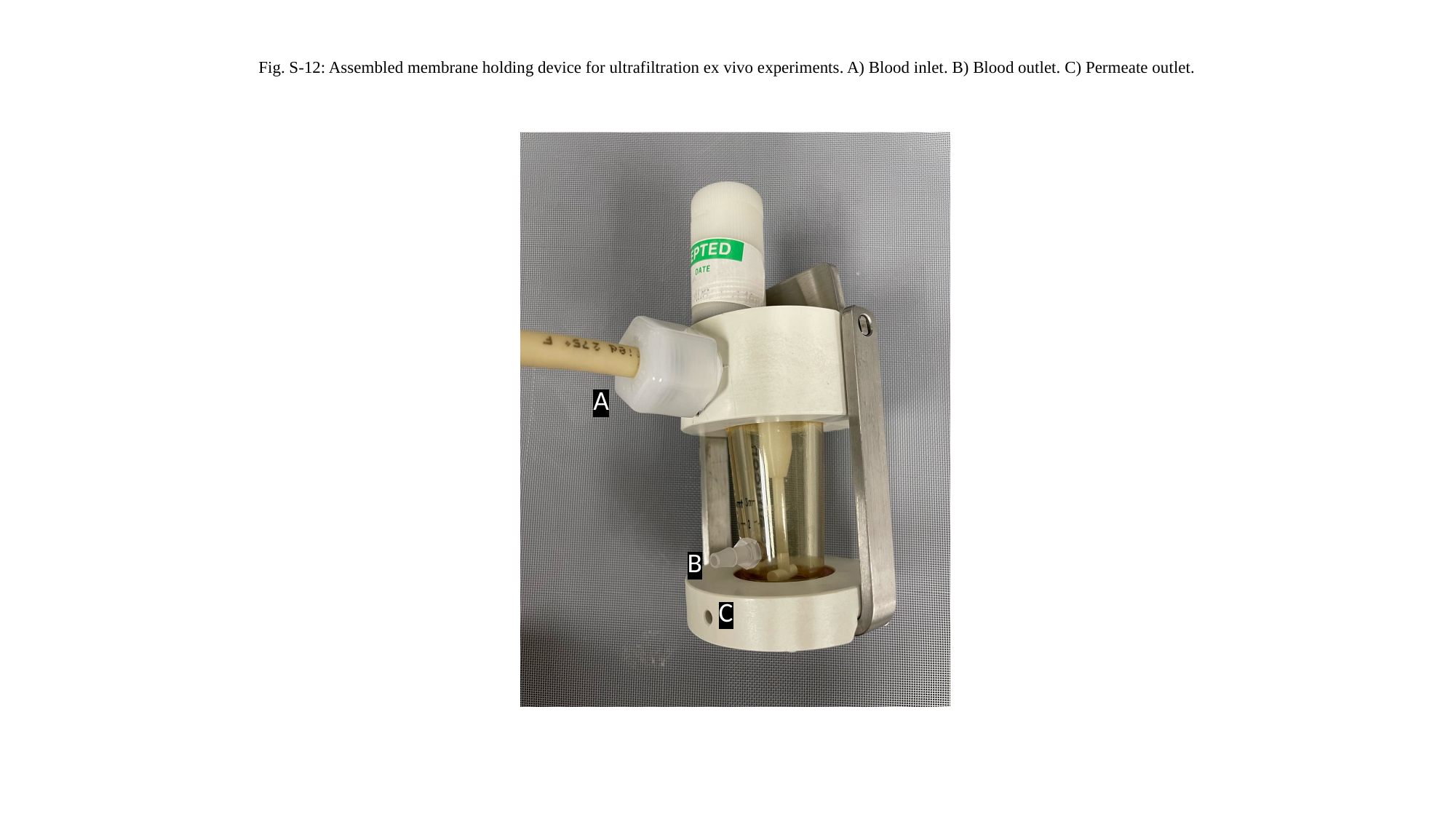

Fig. S-12: Assembled membrane holding device for ultrafiltration ex vivo experiments. A) Blood inlet. B) Blood outlet. C) Permeate outlet.
A
B
C

## Slide 17
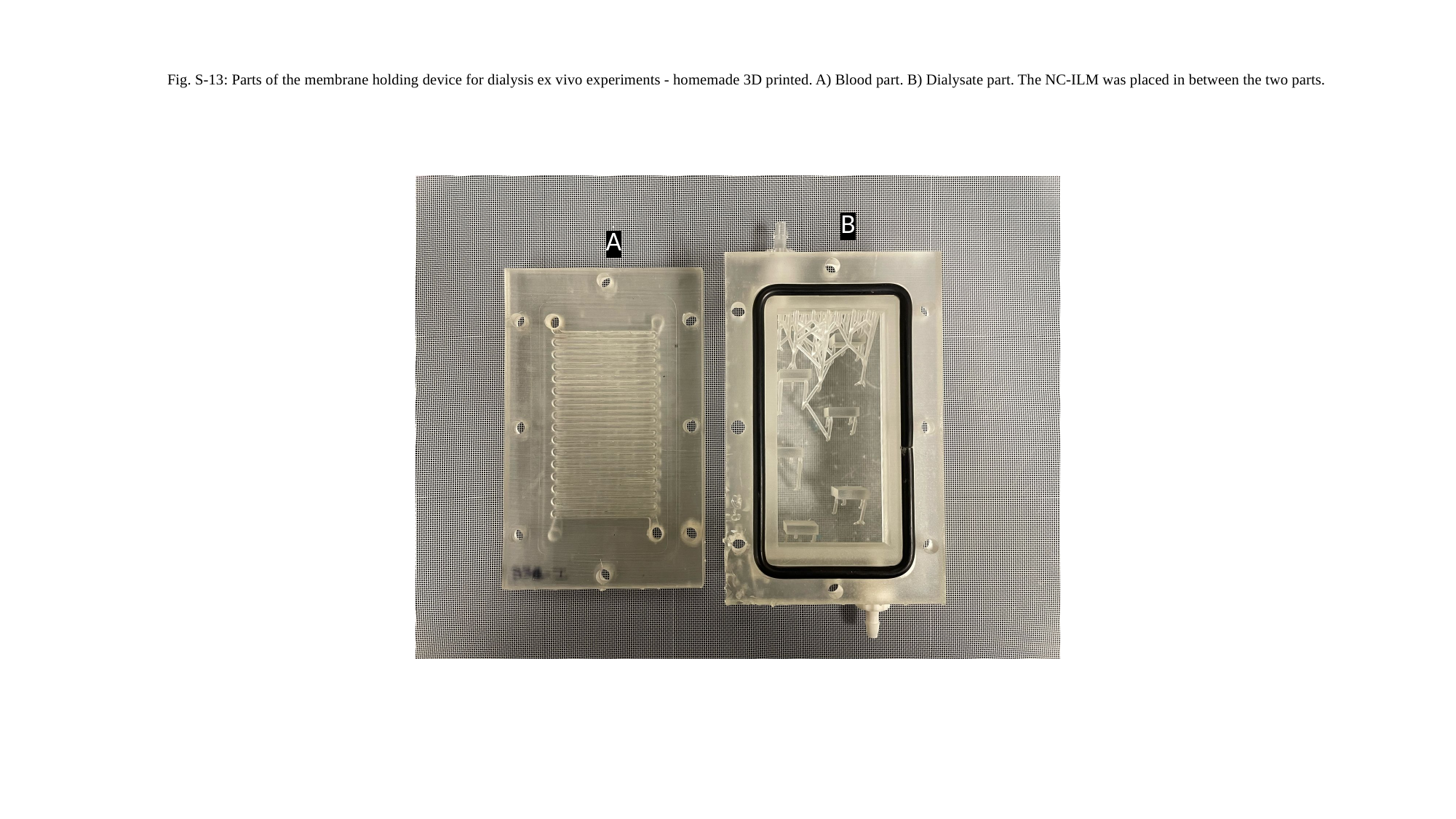

Fig. S-13: Parts of the membrane holding device for dialysis ex vivo experiments - homemade 3D printed. A) Blood part. B) Dialysate part. The NC-ILM was placed in between the two parts.
B
A

## Slide 18
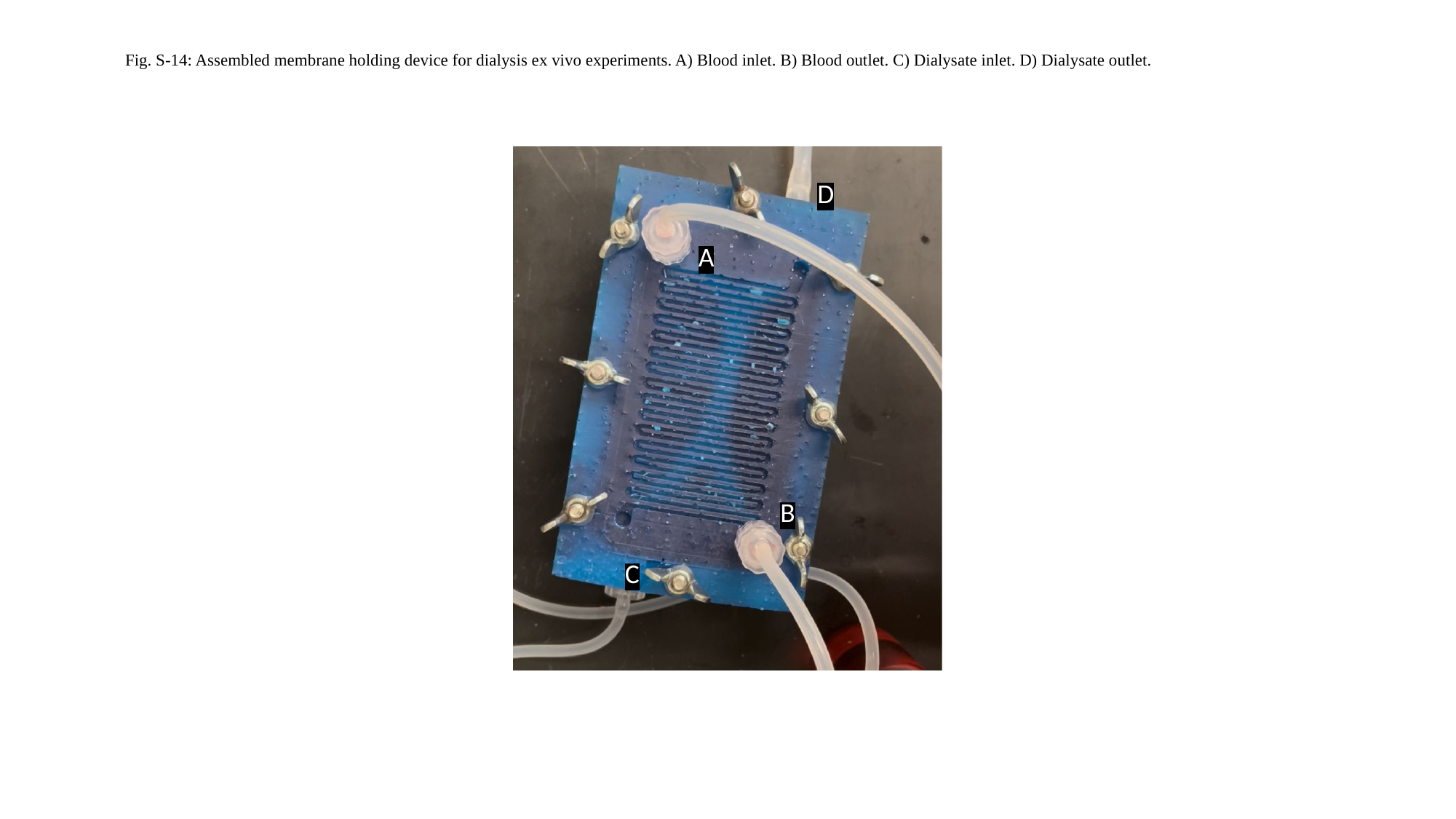

Fig. S-14: Assembled membrane holding device for dialysis ex vivo experiments. A) Blood inlet. B) Blood outlet. C) Dialysate inlet. D) Dialysate outlet.
D
A
B
C

## Slide 19
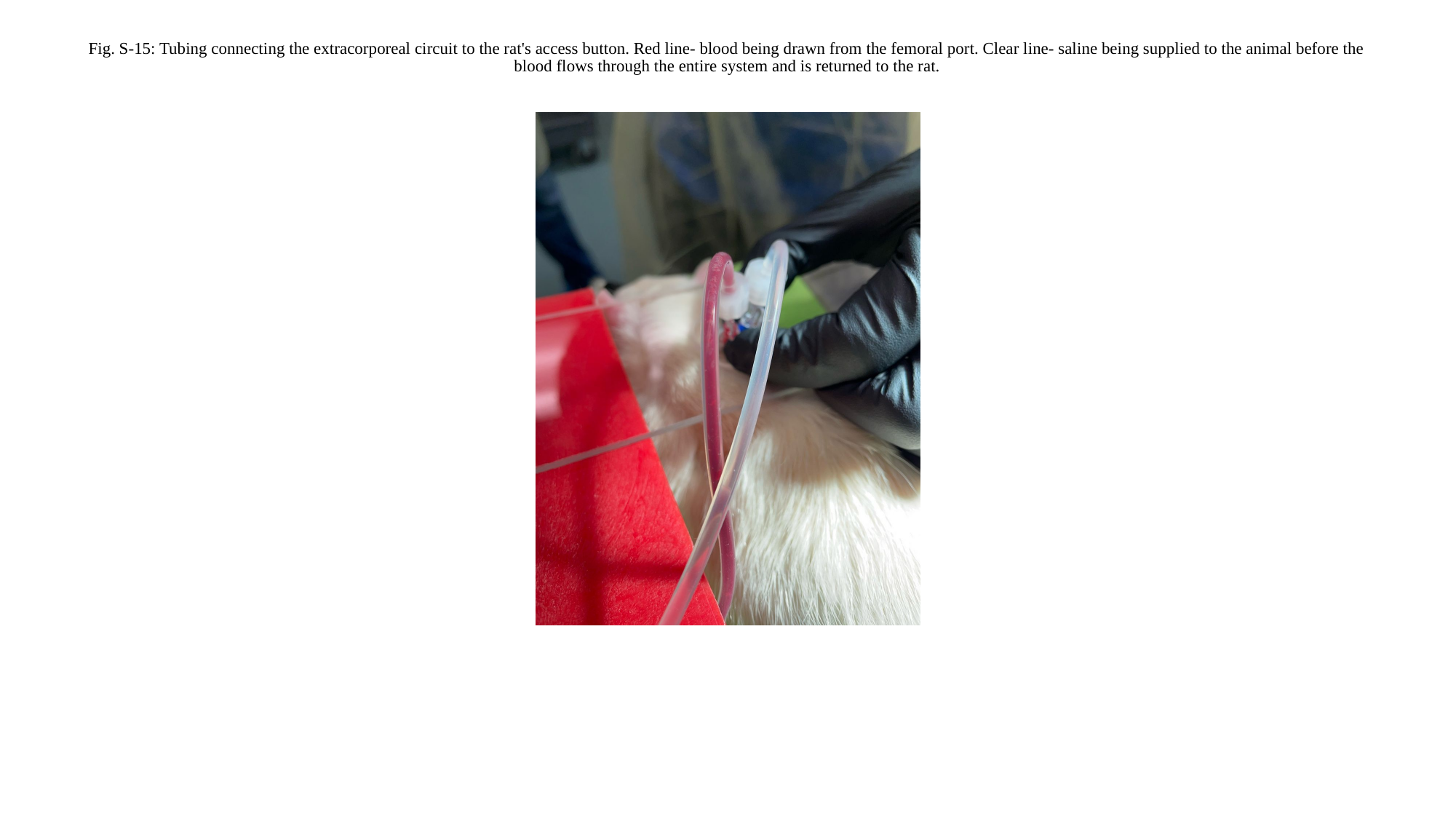

# Fig. S-15: Tubing connecting the extracorporeal circuit to the rat's access button. Red line- blood being drawn from the femoral port. Clear line- saline being supplied to the animal before the blood flows through the entire system and is returned to the rat.

## Slide 20
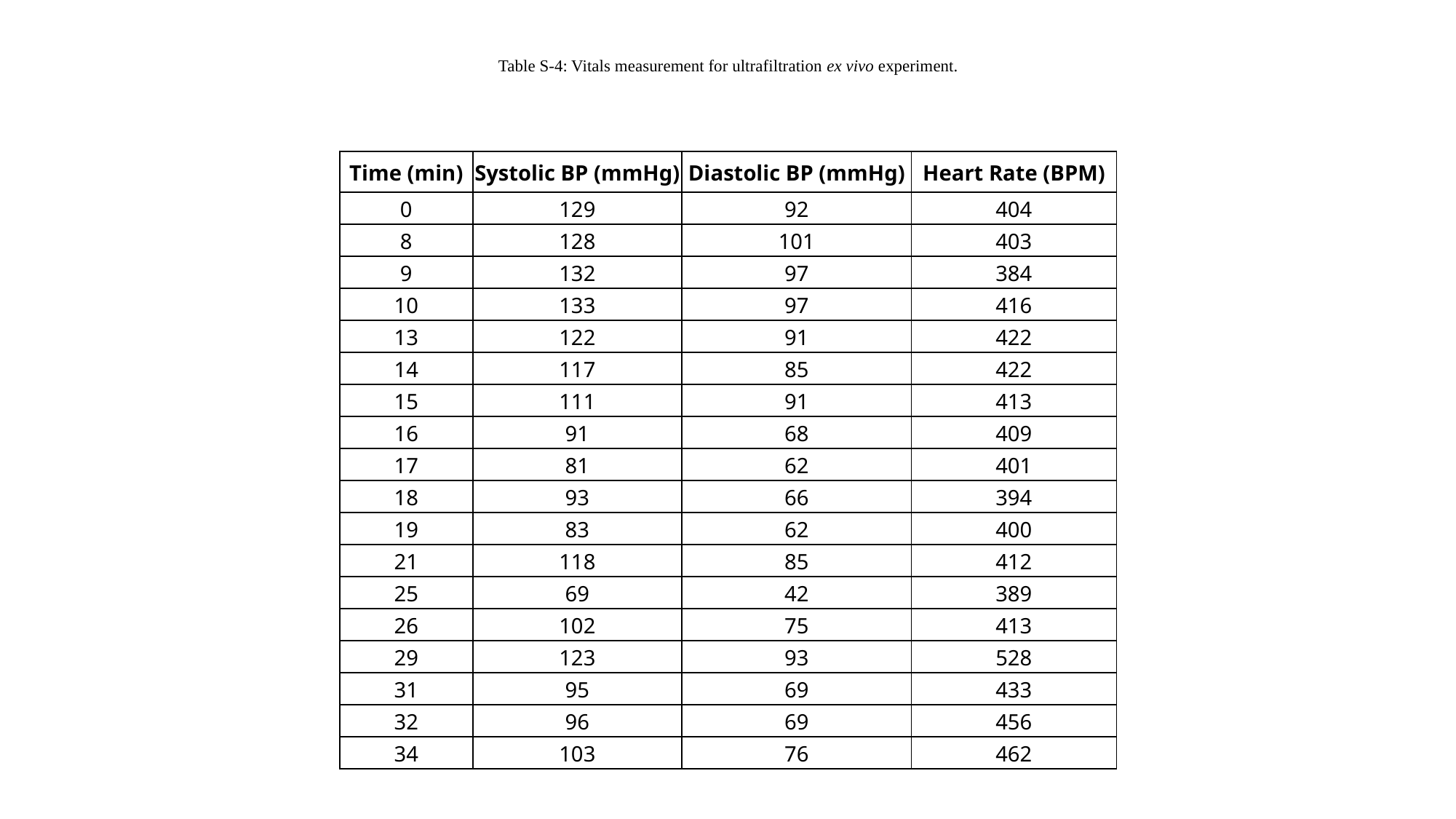

Table S-4: Vitals measurement for ultrafiltration ex vivo experiment.
| Time (min) | Systolic BP (mmHg) | Diastolic BP (mmHg) | Heart Rate (BPM) |
| --- | --- | --- | --- |
| 0 | 129 | 92 | 404 |
| 8 | 128 | 101 | 403 |
| 9 | 132 | 97 | 384 |
| 10 | 133 | 97 | 416 |
| 13 | 122 | 91 | 422 |
| 14 | 117 | 85 | 422 |
| 15 | 111 | 91 | 413 |
| 16 | 91 | 68 | 409 |
| 17 | 81 | 62 | 401 |
| 18 | 93 | 66 | 394 |
| 19 | 83 | 62 | 400 |
| 21 | 118 | 85 | 412 |
| 25 | 69 | 42 | 389 |
| 26 | 102 | 75 | 413 |
| 29 | 123 | 93 | 528 |
| 31 | 95 | 69 | 433 |
| 32 | 96 | 69 | 456 |
| 34 | 103 | 76 | 462 |

## Slide 21
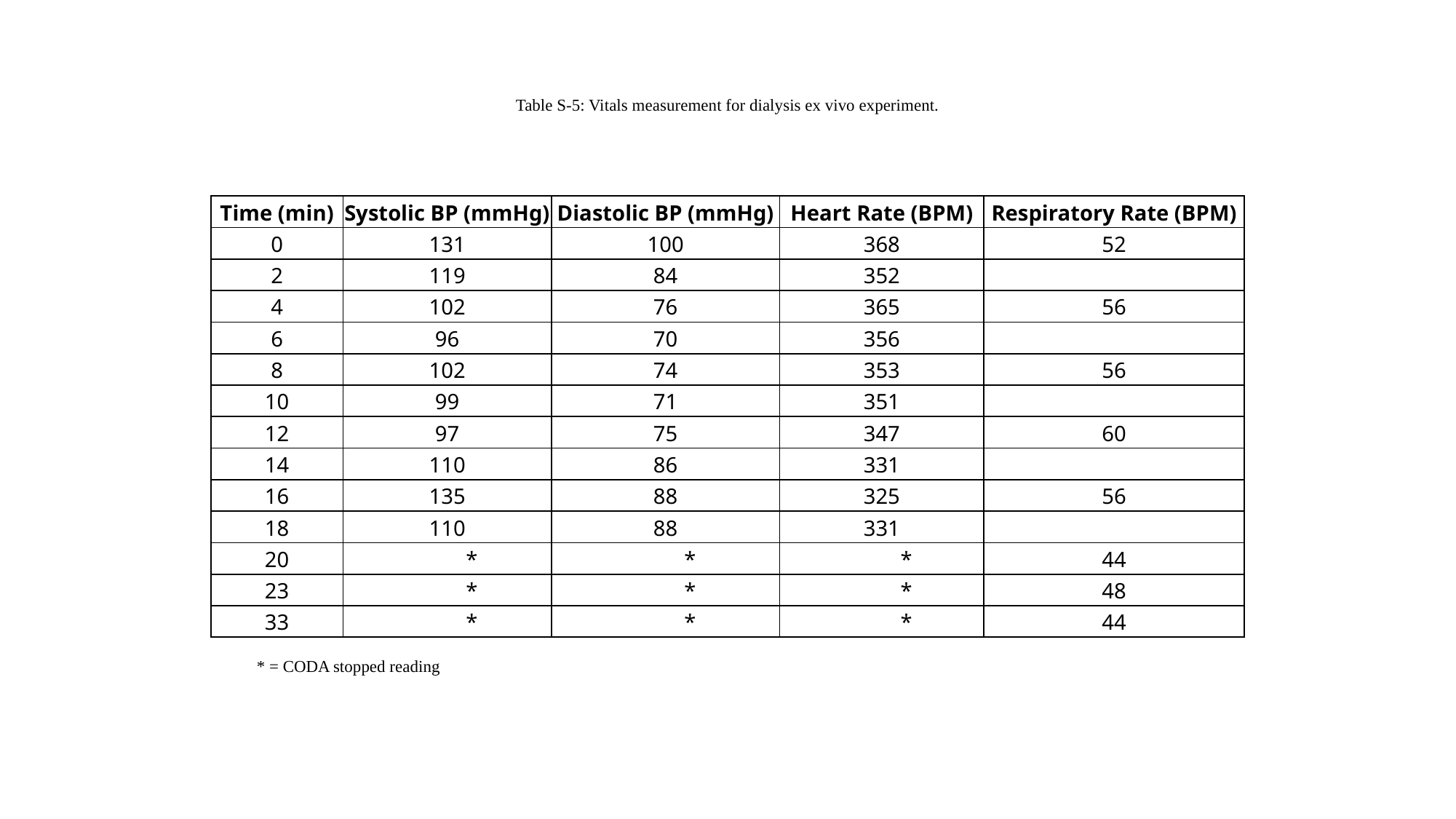

Table S-5: Vitals measurement for dialysis ex vivo experiment.
| Time (min) | Systolic BP (mmHg) | Diastolic BP (mmHg) | Heart Rate (BPM) | Respiratory Rate (BPM) |
| --- | --- | --- | --- | --- |
| 0 | 131 | 100 | 368 | 52 |
| 2 | 119 | 84 | 352 | |
| 4 | 102 | 76 | 365 | 56 |
| 6 | 96 | 70 | 356 | |
| 8 | 102 | 74 | 353 | 56 |
| 10 | 99 | 71 | 351 | |
| 12 | 97 | 75 | 347 | 60 |
| 14 | 110 | 86 | 331 | |
| 16 | 135 | 88 | 325 | 56 |
| 18 | 110 | 88 | 331 | |
| 20 | \* | \* | \* | 44 |
| 23 | \* | \* | \* | 48 |
| 33 | \* | \* | \* | 44 |
* = CODA stopped reading

## Slide 22
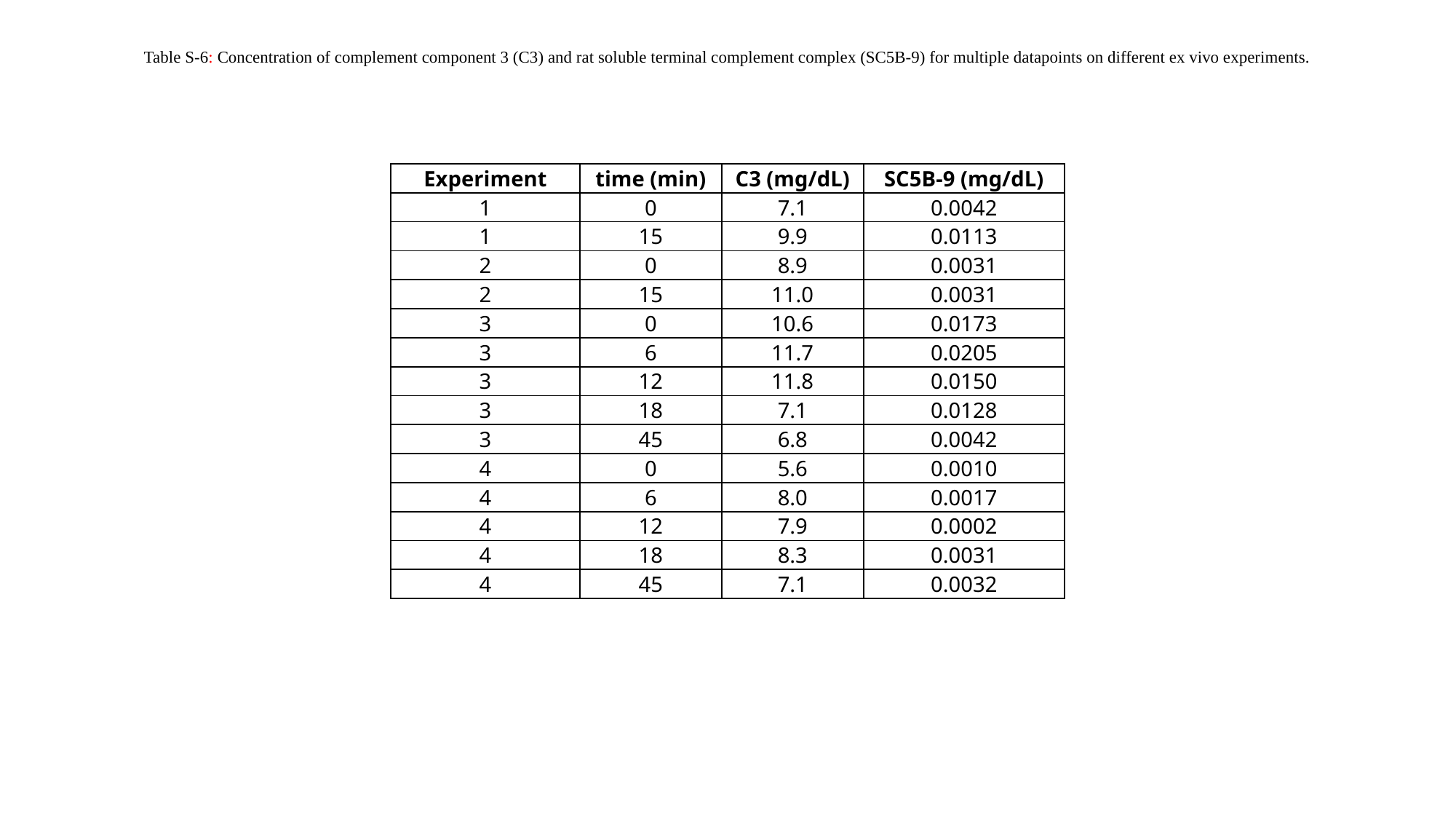

Table S-6: Concentration of complement component 3 (C3) and rat soluble terminal complement complex (SC5B-9) for multiple datapoints on different ex vivo experiments.
| Experiment | time (min) | C3 (mg/dL) | SC5B-9 (mg/dL) |
| --- | --- | --- | --- |
| 1 | 0 | 7.1 | 0.0042 |
| 1 | 15 | 9.9 | 0.0113 |
| 2 | 0 | 8.9 | 0.0031 |
| 2 | 15 | 11.0 | 0.0031 |
| 3 | 0 | 10.6 | 0.0173 |
| 3 | 6 | 11.7 | 0.0205 |
| 3 | 12 | 11.8 | 0.0150 |
| 3 | 18 | 7.1 | 0.0128 |
| 3 | 45 | 6.8 | 0.0042 |
| 4 | 0 | 5.6 | 0.0010 |
| 4 | 6 | 8.0 | 0.0017 |
| 4 | 12 | 7.9 | 0.0002 |
| 4 | 18 | 8.3 | 0.0031 |
| 4 | 45 | 7.1 | 0.0032 |

## Slide 23
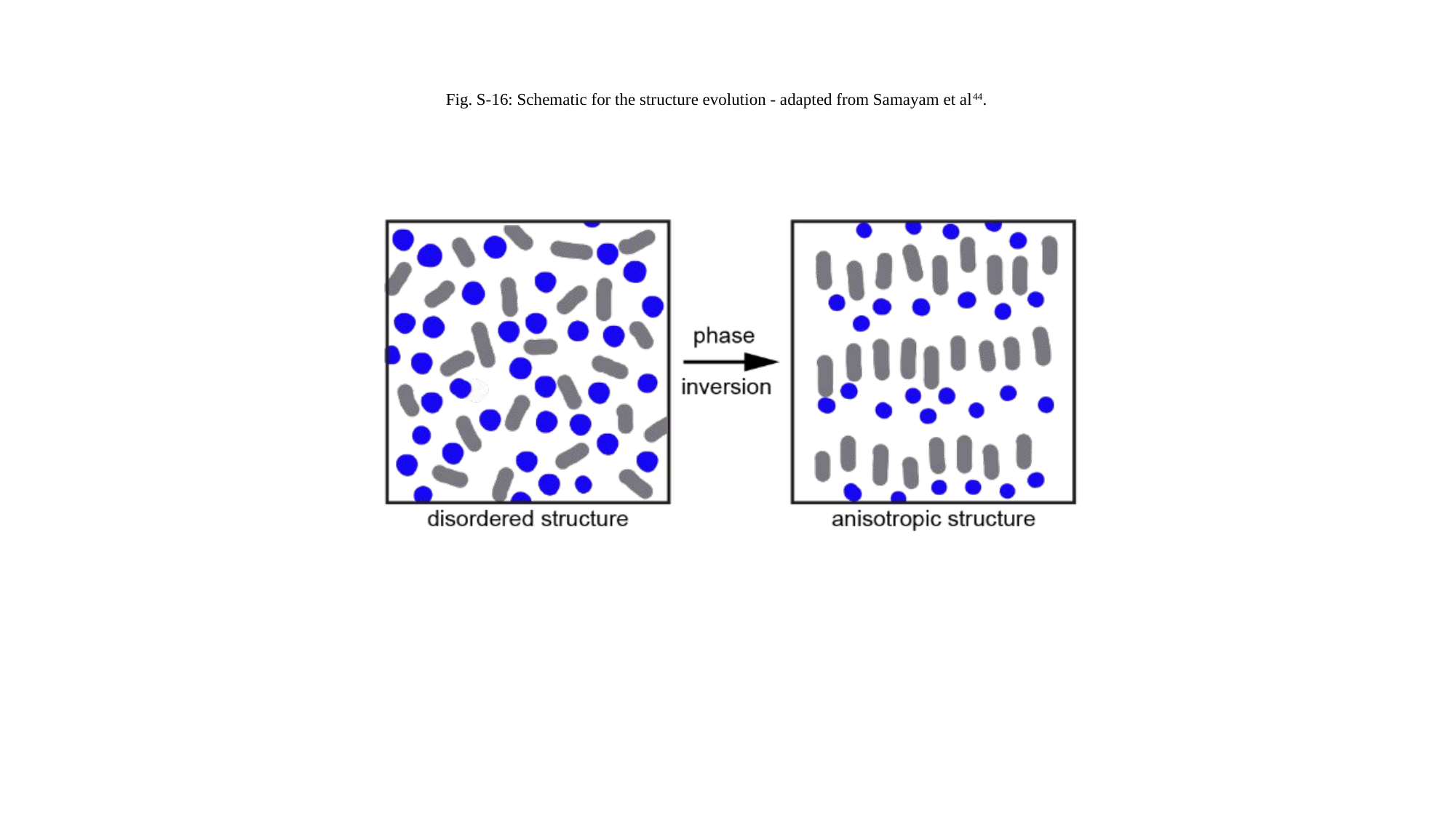

Fig. S-16: Schematic for the structure evolution - adapted from Samayam et al44.
